# A tutorial for estimating Bayesian hierarchical mixture models for visual working memory tasks: Introducing the Bayesian Measurement Modeling (*bmm*) package for R

**DOI:** 10.3758/s13428-025-02643-0

**Published:** 2025-04-14

**Authors:** Gidon T. Frischkorn, Vencislav Popov

**Affiliations:** Department of Psychology, University of Zurich, Zurich, Switzerland

**Keywords:** Tutorial, Mixture model, Visual working memory, Brms, Bayesian modeling

## Abstract

Mixture models for visual working memory tasks using continuous report recall are highly popular measurement models in visual working memory research. Yet, efficient and easy-to-implement hierarchical Bayesian estimation procedures that flexibly enable group or condition comparisons are scarce. Specifically, most software packages implementing mixture models have used maximum likelihood estimation for single-subject data. Such estimation procedures require a large number of trials per participant to obtain robust and reliable estimates. This problem can be solved with hierarchical Bayesian estimation procedures that provide robust and reliable estimates with lower trial numbers. In this tutorial, we illustrate how mixture models for visual working memory tasks can be specified and fit in the newly developed R package *bmm*. The benefit of this implementation over existing hierarchical Bayesian implementations is that *bmm* integrates hierarchical Bayesian estimation of the mixture models with an efficient linear model syntax that enables us to adapt the mixture model to practically any experimental design. Specifically, this implementation allows for varying model parameters over arbitrary groups or experimental conditions. Additionally, the hierarchical structure and the specification of informed priors can frequently improve subject-level parameter estimation and solve estimation problems. We illustrate these benefits in different examples and provide R code for easy adaptation to other use cases.

**Supplementary Information:**

The online version contains supplementary material available at 10.3758/s13428-025-02643-0.

In research on visual working memory, participants are often asked to remember and reproduce continuous features of visual objects such as their color or orientation (Prinzmetal et al., 1998; Wilken & Ma, 2004). These continuous reproduction tasks produce rich data that are often analyzed by mixture measurement models. Using such models allows researchers to dissociate relevant aspects of behavioral performance such as the precision of the memory representation versus the probability of recalling the correct feature (Zhang & Luck, 2008; Bays et al., 2009; Oberauer et al., 2017; Oberauer, 2022). Although mixture models have been widely applied by many researchers in the field,^1^ a flexible, well-documented, and easily accessible means for efficient hierarchical Bayesian estimation of these models is lacking. This tutorial provides an implementation of three highly popular measurement models for visual working memory tasks using the R package *brms* (Bürkner, 2018b) and our newly developed *bmm* R package (Popov & Frischkorn, 2024).

In the continuous reproduction task (sometimes also called delayed estimation task), participants encode a set of visual objects into visual working memory and are then asked to reproduce a specific feature of one cued object on a continuous scale at test (see Fig. 1 for an illustration). Most often, the features used in these tasks are colors sampled from a color wheel (Wilken & Ma, 2004) or continuous orientations of a bar or a triangle (Bays et al., 2011). The set of to-be-remembered objects typically consists of one up to eight objects spatially distributed over the screen. Thus, participants must associate the to-be-remembered features (e.g., color or orientation) with the spatial locations at which they are presented. The precision of the representation of an object’s feature in visual working memory is measured as the angular deviation from the true feature presented at encoding.

**Fig. 1:**
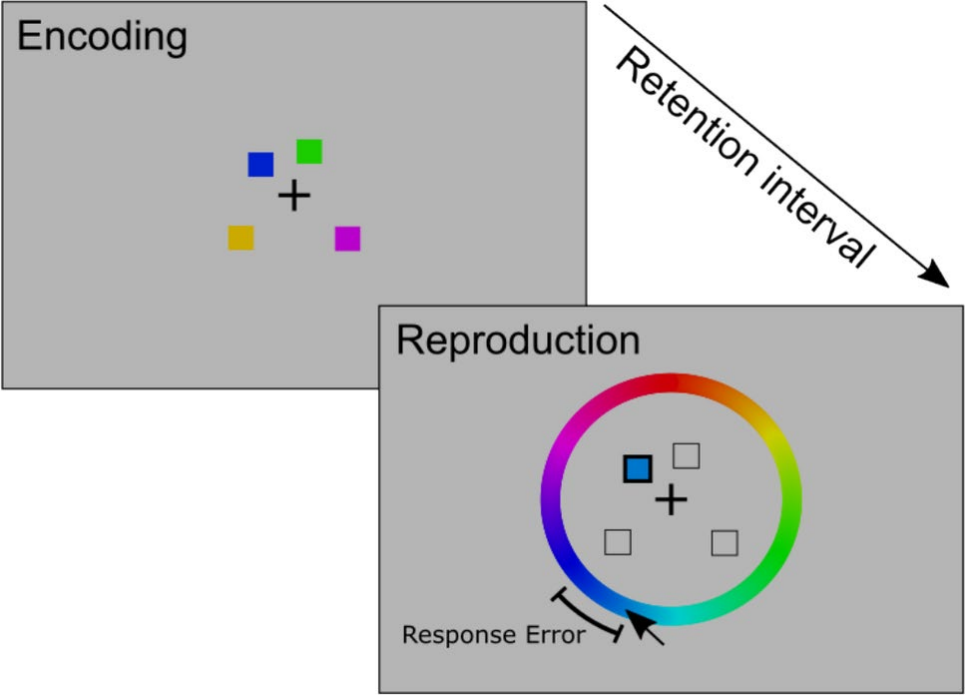
Illustration of a typical continuous reproduction task using colored squares as visual objects. Participants should remember which color was presented at which location, and after a short retention interval, they are asked to reproduce the color of the cued item by selection on the color wheel. The dependent variable is the response error, which is the deviation of the selected response from the originally presented color (illustrated by the arc)

## Benefits of using cognitive measurement models over behavioral performance measures

In such tasks, the simplest measure of performance is the average deviation of the response from the true feature value. In many studies, this average recall error has been the main dependent variable for evaluating the effect of experimental manipulations. Yet, the average recall error confounds different properties of memory representations and does not sufficiently represent the theoretical processes assumed by current models of visual working memory. Therefore, different measurement models have been proposed to formalize distinct aspects of visual working memory models and how they translate into observed behavior (for an overview of currently proposed measurement models, see Oberauer et al., 2017; Oberauer, 2022).

**Fig. 2:**
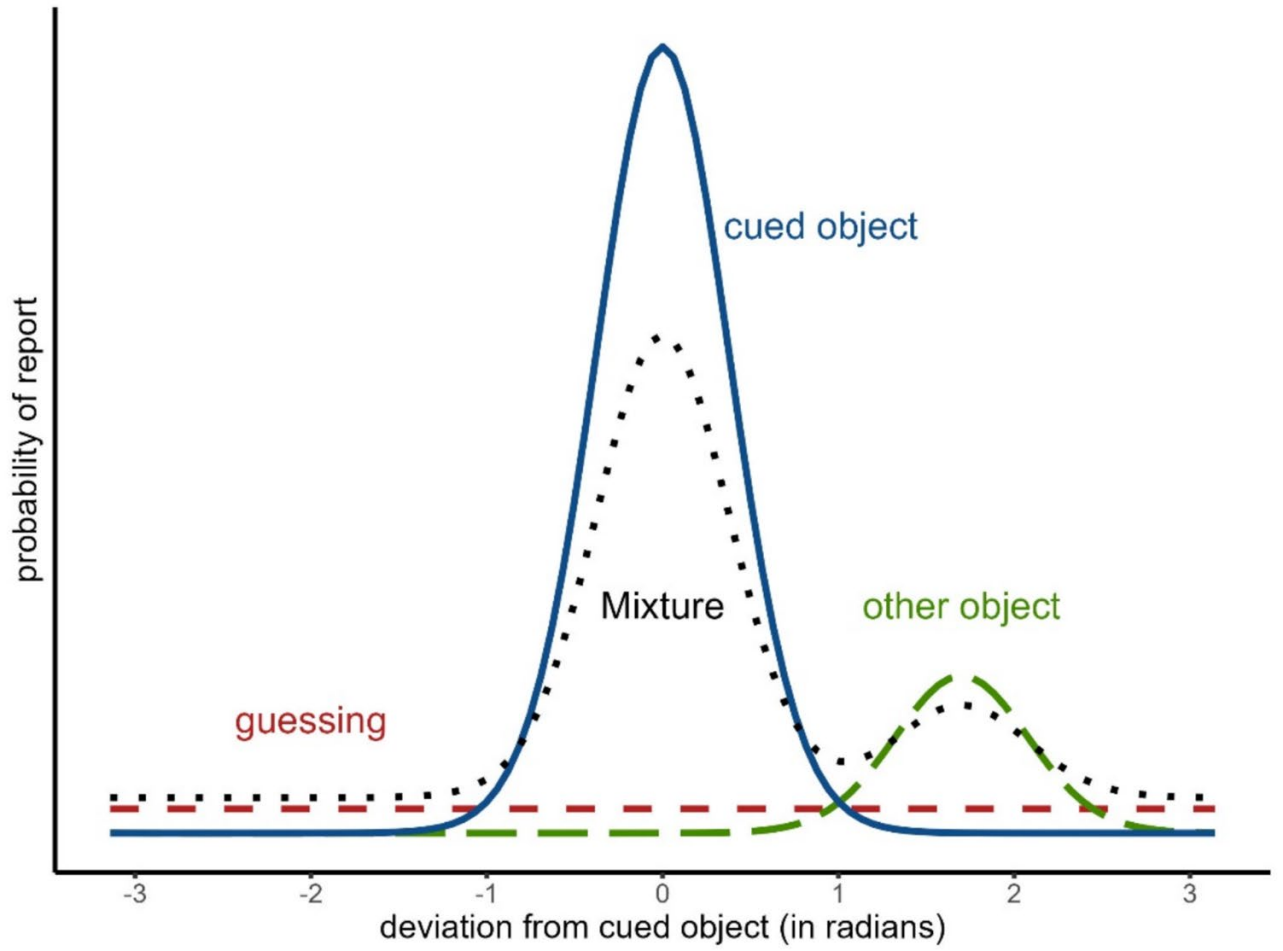
In the described measurement model, the probability of reporting a certain feature value depends on the cognitive state a person is in at recall. If the person recalls the cued object, it will report values following the solid blue distribution. If the person recalls another object, it will report values following the long dashed green distribution centered on the other object value. If the person cannot recall anything, it will be guessing a random value following the dashed red distribution. Depending on the relative proportions of these distributions, the overserved responses will follow the black-dotted mixture of all distributions

Measurement models for the continuous reproduction task provide a more refined representation of memory processes because they decompose the average recall error into several theoretically meaningful parameters. The three measurement models we will be addressing in this tutorial paper are (a) the two-parameter mixture model (Zhang & Luck, 2008), (b) the three-parameter mixture model (Bays et al., 2009), and (c) the interference measurement model (Oberauer et al., 2017). The first two models are mathematically equivalent to constrained versions of the interference measurement model (Oberauer et al., 2017).^2^ At the core of these models is the assumption that responses in continuous reproduction tasks can stem from different distributions depending on the continuous activation of different memory representations or the cognitive state a person is in at recall (see Fig. 2).

The two-parameter mixture model distinguishes two states: (a) having a representation of the cued object with a certain precision of its feature in visual working memory (see solid blue distribution in Fig. 2) versus (b) having no representation in visual working memory and thus guessing a random response (see the dashed red distribution in Fig. 2). Responses based on a noisy memory representation of the correct feature come from a circular normal distribution (i.e., von Mises) centered on the correct feature value, while guessing responses come from a uniform distribution along the entire circle. The three-parameter mixture model adds a third state, namely confusing the cued object with another object shown during encoding and thus reporting the feature of the other object (see long dashed green distribution in Fig. 2). Responses from this state are sometimes called non-target responses or swap errors. Finally, the interference measurement model reformulates these states in terms of different continuous sources of activation—that is, background noise, general, and context activation—and additionally accounts for the spatial proximity between items, predicting that confusion between spatially close items is more likely than between distant items. For all three models, the resulting observed recall distribution is a weighted mixture of the different distributions included in the model (see the dotted black line in Fig. 2).

When applied to the data, the first two mixture models estimate the following parameters: κ, which is the precision of the von Mises distribution of memory representations (which is the inverse of σ, the standard deviation of the circular normal distribution); *p*_mem_, the probability that a response comes from memory of the correct feature; *p*_non-target_, the probability that a response comes from memory of an incorrect feature associated with another object in memory; and *p*_guessing_, the probability that a response is a random guess. In the two-parameter mixture model, *p*_non-target_ = 0 and *p*_mem_ + *p*_guessing_ = 1, while in the three-parameter mixture model *p*_mem_ + *p*_non-target_ + *p*_guessing_ = 1. Formally, according to the mixture models, the probability of responding with a feature *x* is

1 $$P\left(x\right)={p}_{mem}vM\left(x;{\mu }_{1},\upkappa \right)+{p}_{non-target}\frac{\sum_{i=2}^{n}vM\left(x;{\mu }_{i},\upkappa \right)}{n-1}+{p}_{guess}vM\left(x;\text{0,0}\right)$$

where *vM* is the von Mises distribution, ${\mu }_{1}$ is the location of the target feature, ${\mu }_{i}$ represents the locations of the *i* non-target features, and $vM\left(x;\text{0,0}\right)$ is the von Mises distribution with 0 precision, which is equivalent to a uniform circular distribution. Finally, *n* specifies the number of features to be held in memory (i.e., set size).

The interference measurement model (IMM) further decomposes the probabilities of selecting a target, a non-target, or random guessing into continuous sources of activations for context activation *c*, general or item activation *a*, and background noise *b*. In addition, the IMM introduces a generalization gradient that assumes that context cues will generalize dependent on the distance *D*_*j*_ to other items *j* following a Laplace distribution with a slope *s*. This means that the model predicts that swaps to other items that are more similar in context (e.g., are close to the item cued for recall) are more likely than swaps to items less similar in context. Specific details on this decomposition are described by Oberauer et al. (2017) as well as the *bmm* documentation. Formally, the probabilities for recalling the correct item, committing a swap error, or random guessing can be expressed as a function of the IMM parameters:

$$\begin{array}{c}{p}_{mem}=\frac{c+a}{b+\sum_{j=1}^{n}c* {e}^{-s*{D}_{j}}+a}\\ {p}_{swap}=\frac{\sum_{j=2}^{n}c* {e}^{-s*{D}_{j}}+a}{b+\sum_{j=1}^{n}c* {e}^{-s*{D}_{j}}+a}\\ {p}_{guess}=\frac{b}{b+\sum_{j=1}^{n}c* {e}^{-s*{D}_{j}}+a}\end{array}$$

## Benefits of hierarchical Bayesian parameter estimation over non-hierarchical frequentist parameter estimation

Until recently, most researchers have used custom-built code to implement the existing mixture models, in particular the interference measurement model. Although there is software that implements some of these measurement models (e.g., Grange et al., 2021), this software most frequently uses a two-step procedure: First, the parameters of the model are estimated separately for each subject in each condition. Then, in a second step, the parameter estimates are analyzed with traditional inference methods such as *t*-tests, analysis of variance (ANOVA), or linear regression to determine which parameters vary as a function of condition. These methods constrain parameter estimation in several ways and can lead to either the over- or underestimation of standard errors in statistical tests (Boehm et al., 2018; Skrondal & Laake, 2001). Furthermore, to obtain robust parameter estimates, maximum likelihood procedures require many trials per subject and condition (Grange & Moore, 2022; see also Appendix A).

To solve these issues, hierarchical Bayesian implementations of these models have also been proposed (Hardman, 2017; Oberauer et al., 2017; Suchow et al., 2013). Hierarchical Bayesian parameter estimation provides several benefits over frequentist estimations. Critically, by estimating the data from all subjects and all conditions simultaneously, robust parameter estimates can be obtained with less data per subject and condition (Boehm et al., 2018; Oberauer et al., 2017; Vandekerckhove et al., 2011; also see Appendix A for recovery simulations.). Table 1 provides a comprehensive overview comparing non-hierarchical frequentist versus hierarchical Bayesian estimation of these measurement models.

**Table 1:** Comparison of non-hierarchical frequentist versus hierarchical Bayesian estimation of measurement models for visual working memory tasks

	Non-hierarchical frequentist estimation (e.g., using *mixtur***)**	Hierarchical Bayesian estimation using *brms*
Speed of model estimation	Very quick: only seconds per subject	Slow: from a few minutes for simple models to several hours or even days for more complex models and larger datasets
Required data	> 200 retrievals per participant in each condition (Grange & Moore, 2022)	> 50 retrievals per participant in each condition (see Appendix)
Inference	Stepwise approach: (1) Estimate parameters for each subject in each condition, then (2) submit parameters of interest to a statistical test. **Problem:** This approach ignores the uncertainty in parameters in the second step (Boehm et al., 2018)	One-step inference: Parameters for each subject in each condition are estimated in one model. Uncertainty in subject parameters is accounted for in effect estimates of condition differences (Boehm et al., 2018)
Implementation	Simple implementation exists for estimating the model for single-subject data in one condition. For more complex models, researchers must write their likelihood function and adapt it for each experiment	Can be implemented in a well-documented and flexible R package: *brms* Researchers can get support from a broad community using *brms*. The linear model syntax can specify models for almost any use case of the mixture model
Evaluating model fit	Functions implemented in the R package *mixtur* allow one to evaluate model fit For experiments using custom likelihood functions, researchers must write their own functions and scripts to evaluate model fit	Existing tools and functions provided by *brms* provide straightforward methods for checking the convergence of parameter estimates and model fit, and evaluating results
Varying parameters over conditions	Standard implementations estimate model parameters for each subject in each condition. Thus, all parameters must vary over the same set of experimental conditions	The linear model syntax implemented in *brms* specifies which parameter should vary over which condition, and different parameters can be set to vary over different conditions
Model comparison	Separately, for each subject → problem with consistency over the whole sample (for an example, see Popov et al., 2021)	As the model is estimated for the whole sample simultaneously, model comparisons can be made over the whole sample (for an example, see Oberauer et al., 2017)
Possible predictors	Only discrete predictors or group comparisons	Discrete and continuous predictors, as well as group comparisons
Constraints on parameters	If provided by the optimization algorithm, lower and upper bounds on parameters can be specified	The specification of informed priors allows one to impose flexible and meaningful constraints on parameters

Although this comparison oftentimes focuses on frequentist versus Bayesian estimation techniques, the most relevant difference from our perspective is the non-hierarchical versus hierarchical implementation of these models. The hierarchical implementation allows for a one-step procedure and adequately propagates uncertainty through the whole analysis while avoiding biases in the estimation of standard errors for parameters (Boehm et al., 2018).^3^ In principle, this is independent from the estimation algorithm, that is, frequentist maximum likelihood versus Bayesian parameter estimation. However, hierarchical models tend to run into convergence issues when estimated using traditional maximum likelihood estimation, whereas Bayesian methods can alleviate such problems with reasonable priors. Thus, hierarchical models are oftentimes fit to data using Bayesian techniques to avoid convergence issues with high-dimensional parameter spaces.

Why is there a need for an alternative hierarchical Bayesian estimation of mixture models, if these already exist? The proposed implementations have specified the mixture model in JAGS (Oberauer et al., 2017), MATLAB (Suchow et al., 2013), and a no-longer-maintained R package (Hardman, 2017). The JAGS implementation by Oberauer et al. (2017) uses code designed for the specific experimental designs and factors analyzed in Oberauer et al. (2017). Consequently, for other experiments with different factors and factor levels, the JAGS code would need to be adjusted for the specific conditions and groups in it. Thus, researchers would need to adapt JAGS code to apply these models to their specific experiments. This is a skill only researchers with considerable experience in Bayesian modeling have. The MATLAB implementation by Suchow et al. (2013) is more flexible and well documented. However, MATLAB is an expensive proprietary language that not everyone has access to. Nowadays, R is the language of choice when teaching statistical analysis in psychology departments; thus, many more researchers are familiar with it than with MATLAB. Finally, the *CatContModel* R package is not available on *CRAN* and is no longer actively maintained and thus does not provide the stability and flexibility of an actively maintained and tested package such as *brms* and *bmm*.

Furthermore, neither the *MemToolbox* implemented in MATLAB nor the *CatContModel* package allows one to estimate all three models we are presenting implementations for here. They do not provide an implementation of the interference measurement model. And although they provide a hierarchical Bayesian implementation of both the two- and three-parameter mixture models over all subjects, none of the implementations allows one to estimate the three-parameter mixture model or the interference model simultaneously over different set sizes. Specifically, when parameters of the model vary across conditions, these previous implementations need to fit a separate model to each condition and set size, followed by a two-step inference procedure, thus significantly reducing the benefits of previous hierarchical estimation procedures (see Table 2 for a comparison).

**Table 2:** Comparison of *brms*/*bmm* and *MemToolbox*

	*MemToolbox*	*brms/bmm*
Estimation	Bayesian or maximum likelihood	Bayesian
Fitting multiple conditions	Separately to each condition	Jointly (linear model syntax)
Inference over *multiple conditions*	Stepwise approach: (1) Estimate parameters for all subjects in each condition, then (2) submit parameters of interest to a statistical test **Problem:** Ignores uncertainty in parameters in the second step (Boehm et al., 2018). Hierarchical estimation applies only to different subjects, not to different conditions	One-step inference: Parameters for each subject in each condition are estimated in one model. Uncertainty in subject parameters is accounted for in effect estimates of condition differences (Boehm et al., 2018)
Allows continuous predictors	No	Yes
Can fix some parameters across conditions	No	Yes—any model parameter can be predicted by any combination of conditions
Behavioral tasks	Continuous report, change detection	Continuous report, custom (see *General discussion*)
Included models “out-of-the-box”	Two-parameter, three-parameter, variable precision, slot + averaging, slots + resources	Two-parameter, three-parameter, interference measurement model

To overcome these difficulties and to make the discussed measurement models more accessible, we illustrate how to estimate these models using our newly developed *bmm* package and how to implement these models in *brms*, a general-purpose package for estimating Bayesian multilevel regression models (Bürkner, 2017, 2018a, b), without relying on *bmm*. The major benefit of this implementation is that *bmm* provides a powerful linear model syntax that allows us to flexibly specify which model parameters should vary depending on discrete or continuous predictors. Therefore, *bmm* allows a general-purpose implementation of the measurement models for visual working memory tasks that can be adapted to practically any experimental design. Additionally, *bmm* uses the probabilistic programming language Stan (Carpenter et al., 2017) to estimate parameters. Arguably, Stan is the most cutting-edge estimation algorithm for Bayesian modeling and provides robust parameter estimates even when parameters are correlated and with a small posterior sample size. Finally, *bmm* seamlessly integrates with *brms*, a general-purpose package for Bayesian regression models that has a large and active community, which can assist in solving problems. In sum, our implementation of measurement models for visual working memory tasks in *bmm* will enable more researchers to use these models in their work and will provide state-of-the-art hierarchical Bayesian estimation for them**.**

### *brms* versus *bmm*

All mixture models presented in this tutorial can be implemented in *brms* without the need for additional packages or functions.^4^ Implementing the simple two-parameter mixture model (Zhang & Luck, 2008) this way is relatively easy. However, implementing the three-parameter mixture model (Bays et al., 2009) and the interference measurement model (Oberauer & Lin, 2017) is more complicated, because they require transformation of parameters and combining multiple parameters into one.^5^ Additionally, we implemented additional functionality to allow the estimation of both the three-parameter mixture model and the interference measurement model over varying set sizes. To make these implementations more accessible and reduce the chance for errors, we developed the Bayesian Measurement Modeling (*bmm)* R package, which provides wrapper functions around *brms* that take care of these procedures and allow the user to specify the desired model more easily.^6^

In the examples that follow, we demonstrate how the measurement models can be implemented in *bmm*. We separately provide information on how to extract all the information *bmm* is compiling to run these models in *brms* in the section *Customizing and extending models implemented in bmm*, so that readers can become familiar with how the models are implemented and how they can potentially adapt them. In the first example, we additionally introduce basic concepts regarding the parameterization of the measurement models that will help researchers better understand how parameters are estimated and should be interpreted.

## Example 1: Estimating the two-parameter mixture model in *bmm*

Estimating the two-parameter mixture model in *bmm* is designed to be easy and accessible. In fact, it takes only a few lines of code to set everything up to fit the model:
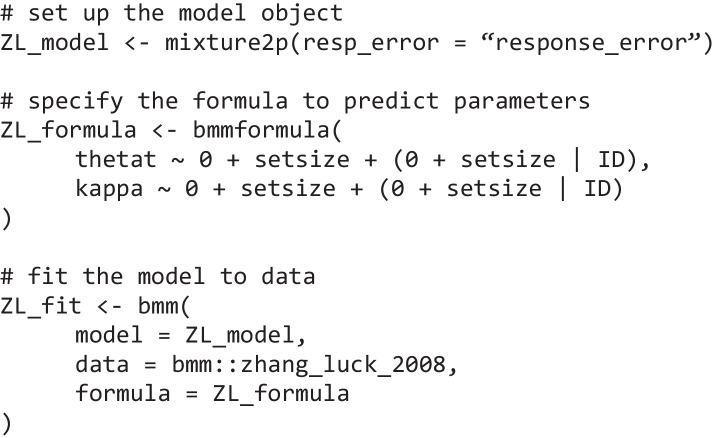


The three basic steps that *bmm* requires are (1) setting up the model object and providing the variable names that contain critical information for identifying the model, (2) specifying a *bmmformula* to predict the parameters of the model, and (3) calling the *bmm* function to fit the model to data using the specified formula. The next section explains in more detail what *bmm* implements internally in each of these steps and introduces some conceptual underpinnings of the measurement models. When using *bmm*, you do not have to perform these steps yourself; nevertheless, we wanted to provide some background on how these models are set up within the *bmm* package.

## Setting up a mixture model in *brms*

In most common *brms* use cases, researchers specify how the dependent variable is distributed (e.g., a Gaussian distribution for continuous data or a binomial distribution for accuracy data) and then specify a linear model that predicts parameters of this data distribution (usually the mean or location parameter) depending on categorical or continuous predictors. Additionally, the model syntax implements generalized hierarchical modeling, meaning you can specify random effects over grouping variables, such as subjects or items, that account for variability between the different group members in the overall fixed effect. Initially*, brms* was not specifically designed to implement mixture models for visual working memory, but one of its recent updates makes that possible: in addition to linear models for single data distributions, *brms* now also allows users to specify mixtures of data distributions. This is a critical feature that we built on for estimating the above-described measurement models for visual working memory tasks. Specifically, there are five steps we need to take for estimating these mixture models:

1. Specify the mixture family to use for the measurement model.
2. Specify the model formula to set up the model and predict parameters of interest.
3. Set priors to follow the assumptions of the different measurement models and properly identify the different mixture components.
4. Estimate the model.
5. Evaluate model fit and results.

The *bmm* package takes care of all these steps for you, so that you can focus on the details specific to your experiment, namely, (a) how relevant variables for the model are labeled in your data, and (b) which parameters should vary as a function of experimental manipulations. To additionally showcase some of the powerful features of this implementation in *bmm* we will give some examples for different experimental designs. The section of this first example is long because we explain all concepts in detail, but as you have seen above, the actual code required to fit the model is short and simple. In fact, setting up most of the examples and running the models took us from as little as 30 min to less than a few hours, showcasing the flexibility and adaptability of the *bmm* implementation of the mixture models.

## Setting up a model object and specifying relevant variables

**Fig. 3:**
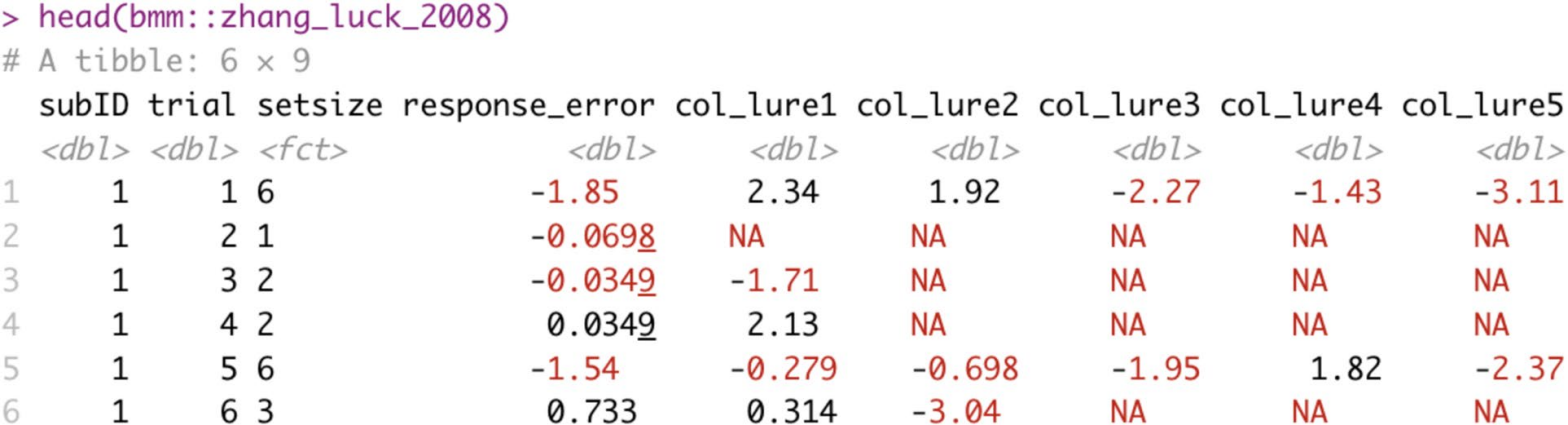
Structure of the Zhang and Luck (2008) dataset included in the *bmm* package. subID = participant number; trial = trial number; setsize = number of presented colors; response_error = difference between response and target color location in radians; col_lure1 to col_lure5 = location of non-target colors relative to the target color location (in radians)


For our first example, we used the data from Experiment 2 reported in Zhang and Luck (2008) included as an example dataset in the *bmm* package. This experiment included a varying number (1, 2, 3, or 6) of spatially distributed colored squares on the screen, and participants were asked to report the color of one randomly chosen square after a short retention interval on the color wheel. For modeling with *bmm,* the data need to be in long format, where each row represents a single observation and each column specifies what conditions generated this observation (see Fig. 3 for an illustration of the first few rows of the dataset). To link the response variable in our data to the model, we set up a *bmmodel* object. This serves two purposes: first, it declares which model we want to fit to the data, and second, it tells *bmm* which variables contain the relevant response variables and additional variables that are required to identify the model. In the case of the two-parameter mixture model, the only required variable is the response error, so setting up the *bmmodel* object is very simple:

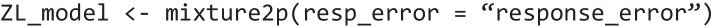


Two points need to be considered when preparing the data: (1) the von Mises distribution implemented in *bmm* is scaled to radians, so the response variable needs to be converted to radians if it was originally coded in degrees (the *bmm* package includes a *deg2rad* function for this purpose), and (2) the response variable refers to the response error, that is, the deviation of the response in one trial from the target response of the to-be-reproduced item.

## Specifying the mixture family

For this first example, we estimate the two-parameter mixture model, allowing the precision of memory and the probability that an item is in memory to vary as a function of set size. To specify the mixture family required for the two-parameter mixture model, *bmm* internally uses the mixture function provided by *brms*.^7^ For the two-parameter mixture model, we need a mixture of two distributions, one for guessing and one for sampling from the memory representation. To ensure that both distributions cover the same range of responses, *bmm* specifies a mixture of two von Mises distributions. This is possible because a von Mises distribution with a precision of zero is equal to a uniform distribution over the circular space. The *brmsfamily* specified by *bmm* is stored in the fit object returned by the *bmm* function and can be accessed via the family element of the *bmmfit* object returned when a model is fitted: ZL_fit$family. The mixture family is specified by calling the mixture function from *brms* and passing the distributions the mixture should contain:
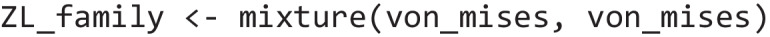


For additional information on how to extract the information compiled by *bmm*, see the section *Customizing and extending models implemented in bmm*.

## Explanation of model parameters and specifying the model formula

Next, we need to specify the model formula to predict parameters from the model. In *bmm*, this is done by using the *bmmformula* (or short *bmf*) function. The aim of this function is that users only need to specify the linear model formulas for the model parameters, while *bmm* internally specifies all additional formulas that are needed to set the model up for *brms*. For the mixture models implemented in *bmm*, there are two classes of parameters: (a) distributional parameters of each distribution contained in the mixture (such as the mean, *mu,* and the precision, *kappa,* of the von Mises distributions), and (b) mixing proportions (*theta*) for each distribution of the mixture.

It is important to note that *bmm* does not directly estimate the probabilities that each response comes from one of the distributions (e.g., *p*_mem_ and *p*_guessing_). Instead, *bmm* estimates mixing proportions *θ* that are weights applied to each of the mixture distributions, which are transformed into probabilities (e.g., *p*_mem_ and *p*_guessing_) using a *softmax* normalization. Thus, for a mixture of *K* distributions, the probability for the data to stem from a specific mixture distribution *i* is

2 $${p}_{i}=\frac{{e}^{{\theta }_{i}}}{{\sum }_{j =1}^{K}{e}^{{\theta }_{j}}}$$

Therefore, the mixing weights can range from minus to plus infinity, with negative values resulting in a low probability of the data stemming from the respective mixture distribution and positive values resulting in high probabilities of the data coming from the respective mixture distribution. Because the distribution probabilities sum to 1, there would be infinitely many solutions for obtaining specific probabilities if all mixture weights were estimated (e.g., for any value *j*, if ${\theta }_{1}={\theta }_{2}=j$, *p*_*mem*_ = 0.5). Thus, by default, one of the mixing proportions is fixed to 0 (usually the mixing proportion of the guessing distribution), and all other proportions are estimated freely. For example, if the mixing weight for responses coming from memory is estimated as 2, we can obtain the response probabilities as such:

$$\begin{array}{c}{p}_{mem}= \frac{{e}^{{\theta }_{1}}}{{e}^{{\theta }_{1}}+{e}^{{\theta }_{2}}}=\frac{{e}^{2}}{{e}^{2}+{e}^{0}}=\frac{7.389}{7.389+1}=0.88\\ {p}_{guess}= \frac{{e}^{{\theta }_{2}}}{{e}^{{\theta }_{1}}+{e}^{{\theta }_{2}}}=\frac{{e}^{0}}{{e}^{2}+{e}^{0}}=\frac{1}{7.389+1}=0.12\end{array}$$

In *brms*, both the distribution parameters and the mixing proportions are indexed with an integer which specifies the distribution they are associated with. When using a mixture of two von Mises distributions, we thus have two sets of the distributional parameters: *mu1* and *mu2* for the means, and *kappa1* and *kappa2* for the precision. Additionally, we have two mixing weights *theta1* and *theta2*. Distribution 1 is the von Mises distribution centered on the target response, while distribution 2 is the uniform circular distribution. Technically, there are thus six parameters in this model. Practically, four of these parameters will be fixed to a constant or determined by some variable in our data.^8^ This is all done internally by *bmm*; thus, only two parameters remain to be estimated. Furthermore, to keep the parameter labels consistent across models, *bmm* internally relabels these parameters to *kappa* (the memory precision) and *thetat* (mixing proportion for responses coming from memory, so-called *target* responses).

Thus, for specifying that both the probability of recalling an item from memory (i.e., thetat) and the memory precision (i.e., kappa) should vary over the different set sizes, we can specify the following *bmmformula* (or in short, *bmf*):
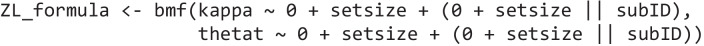


For all linear model formulas in *bmm*, the left side of an equation refers to the to-be predicted parameter and the right side specifies the variables used to predict it. In this formula, the precision of memory responses (*kappa*) is set to vary over *setsize*, and additionally this *setsize* effect can vary over subjects indexed through the *subID* variable. Likewise, the mixing proportion of the von Mises distribution of the target responses (*thetat*) varies over the *setsize* variable, and this effect can also vary over subjects. Specifically, using the 0 + *setsize* coding, we suppressed the estimation of an intercept and directly estimated the parameter values for each set size. Instead, we could have used an effect coding specifying 1 + *setsize*. Then, *bmm* would have estimated an intercept and effects following the effect coding of the *setsize* variable (e.g., treatment or sum-to-zero contrast as specified by *contr.treat* or *contr.sum* in R). The random slopes for the *setsize* effects are set by specifying which of the fixed effects can vary over which grouping variable using the || coding. The double vertical line additionally specifies that correlations between the different random effects should not be estimated. This setting speeds up model estimation and makes interpretation of the fixed effects more straightforward. 

Should you be interested in accessing the full formula that *bmm* generates to estimate the model in *brms*, you can access it via the *bmmfit* object. The formula element (i.e., ZL_fit$formula) will return the full formula compiled by *bmm* and allows you to adapt the model for your own purposes. We will provide further details on customizing these models in the section *Customizing and extending models implemented in bmm.*

## Setting priors for the model

To sufficiently identify the mixture distributions and implement assumptions from the respective measurement model, *bmm* internally constrains some model parameters via priors. Ultimately, in the two-parameter mixture model, we only estimate (a) the precision of the target distribution, i.e. *kappa*, and (b) the probability of an item being stored in memory, that is, defined by the mixing proportion *thetat*. All other model parameters are internally constrained via priors or through the data.

**Fig. 4:**
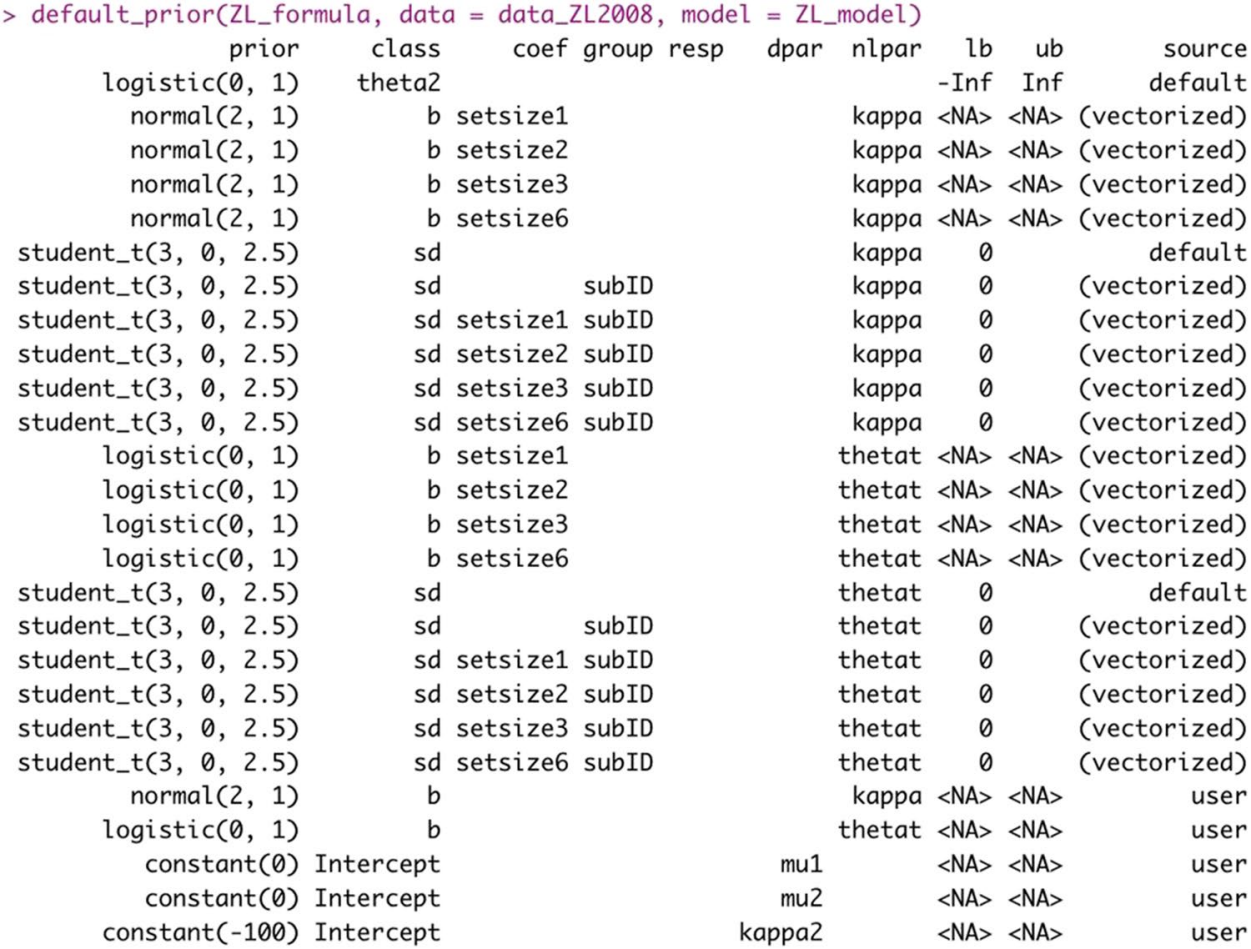
Example of a call of the default_prior function and its generated output

Additionally, *bmm* sets default priors for each model. This is done to improve convergence of parameter estimation and speed up sampling. You can access the priors with the *default_prior* function that can also be used for *brms* models (see Fig. 4). This function takes the specified *bmmformula*, the *data*, and the *bmmodel* object as input and returns a data frame with the priors specified internally by *bmm*. If you want to specify custom priors for your model, we recommend running this function first, to see which priors are specified for the model by default. If you want to change these priors, you can specify your own priors using the *prior* function implemented in *brms* and pass these priors when fitting the model using the *bmm* function.

The priors on the estimated parameters *kappa* and *thetat* make up most of the specified priors in Fig. 4. In addition, we want the guessing distribution to be uniform over the whole circular space. This is achieved by fixing the precision of the second von Mises distribution (i.e., *kappa2*) to practically zero. For estimation purposes, *brms* uses a logarithmic link function for the precision parameters of the von Mises. Thus, the precision *kappa2* on the native scale is transformed onto the parameter space using a logarithmic function. As *log(0)* is not defined, we fix kappa for the second von Mises to a value very close to zero. Practically, any native kappa value below 10^−3^ achieves a virtually uniform distribution.^9^ Since the priors are set on the transformed parameters, this would equate to any value smaller than ${\text{log}(10}^{-3})$. For convenience, we set it to − 100, which is much smaller. Naturally, the mean or location of a uniform distribution on a circular space is not properly defined; thus, we must also fix this value (*mu2*), conventionally to 0. Likewise, the location of our target distribution (*mu1*) is fixed. For all examples, we have specified the model formula using the response error as dependent variable. Thus, the location of the memory distribution is fixed to zero.^10^

Finally, the *softmax* transformation needs to be identified by fixing one mixture proportion as a reference. This is internally implemented in *brms* for all mixture models. With this default, the freely estimated mixture proportion for any mixture of two distributions can be transformed into mixture probabilities using the inverse logit function. For more than two distributions, we need to compute the probability using the *softmax* normalization. However, this normalization is implemented in *bmm*, or it can be computed manually.

## Estimating the model

Using this specification, we can now estimate the parameters of the model. This is done using the Bayesian measurement model function, bmm, of the *bmm* package, as in the example at the beginning of this section. We need to pass the specified *bmmodel*, the *bmmformula*, and the data. All the rest is set up internally: 
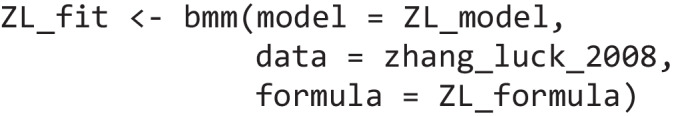


Additionally, you can pass further arguments to the function: for example, how many warmup and total iterations should be performed, or how many MCMC chains should be sampled. For an explanation of these additional arguments, please see the *bmm* and *brms* user guide.

When *bmm* is called, the function will first perform some checks to verify that all information is valid, then it will compile the code of the Stan model, and finally, after finishing compilation, it will run the specified number of Markov chain Monte Carlo (MCMC) chains for parameter estimation. The default value is four chains, with each having 1000 warmup samples and 1000 samples after warmup. For faster estimation, we recommend setting parallel sampling by specifying the number of cores used for sampling using the cores argument. Furthermore, to avoid having to re-estimate the model each time after you close an R session, consider saving the *bmmfit* object using the file argument. For an example of how to set up your R code to only estimate the model should there not be a saved file for the model object, see the GitHub repository that provides R code and results for all examples.

## Evaluating convergence, model fit, and results

Once parameter estimation is completed, we need to evaluate the convergence of the MCMC chains, the model fit, and results from the parameter estimation. For this, we can choose from the wide range of functions provided by *brms* and other R packages designed for analyzing posterior predictive samples from Bayesian models (*bayesplot*, *tidybayes*, etc.).

**Convergence** Stan (Carpenter et al., 2017), the probabilistic language used for sampling in *bmm*, will automatically print warning messages at the end of sampling should it detect problems that can point towards convergence issues. Typically, these are either the occurrence of *divergent transitions* during sampling, reaching the *maximal treedepth* during sampling, or R-hat values larger than 1.05. Generally, the models implemented in *bmm* are set up to provide robust and reliable parameter estimation and thus avoid such warnings as much as possible. Nonetheless, in some cases the combination of little data and certain model assumptions can lead to sampling problems. We will not elaborate on the details of such problems here, as this is beyond the scope of this tutorial. Generally, you can change the behavior of the sampler and avoid these warnings by passing a list of options when calling the *bmm* function. You can find more information on such warnings and how to address these on the Stan webpage: https://mc-stan.org/misc/warnings.html.

**Fig. 5:**
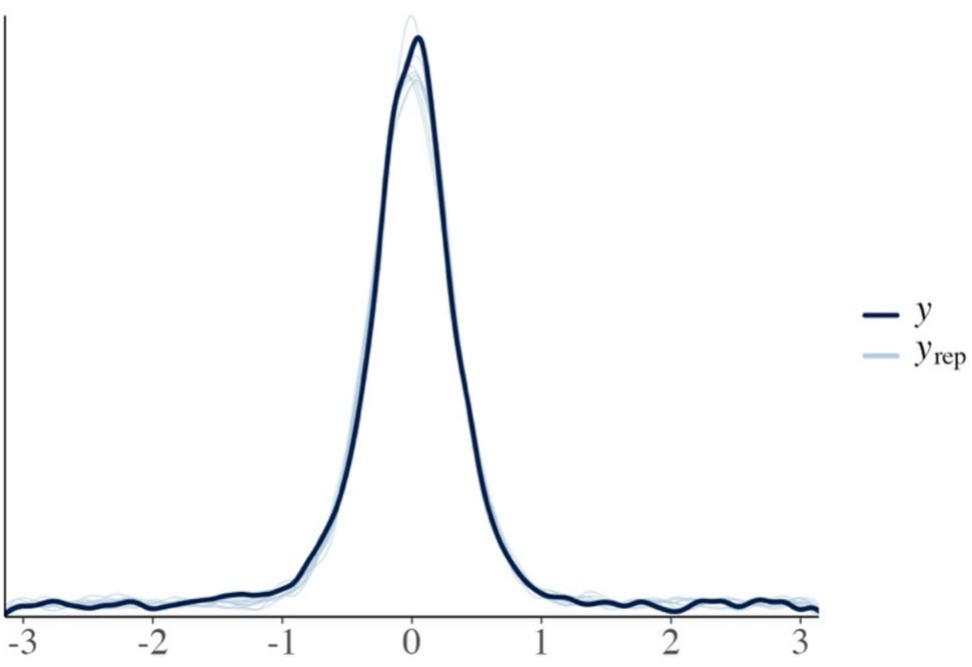
Example of a posterior predictive plot obtained via the pp_check function provided by *brms*. The black line illustrates the distribution of the data. The different blue lines illustrate ten independent predicted distributions from the model. The better the overlay of the model-predicted distributions with the data, the better the model captures the data. The posterior predictive plot thus illustrates a good fit of the model to the data

**Model fit** With respect to model fit, our recommendation is to at least have a look at graphical plots in which posterior predictions from the model are overlaid on the observed data. As *bmm* feeds seamlessly into *brms*, this can be done using the pp_check function implemented in *brms* (see Fig. 5 for an illustration). The model fits the data well, if the observed data distribution (black line in Fig. 5) is closely overlaid with the distribution of the posterior predicted responses (light blue lines in Fig. 5).

**Fig. 6:**
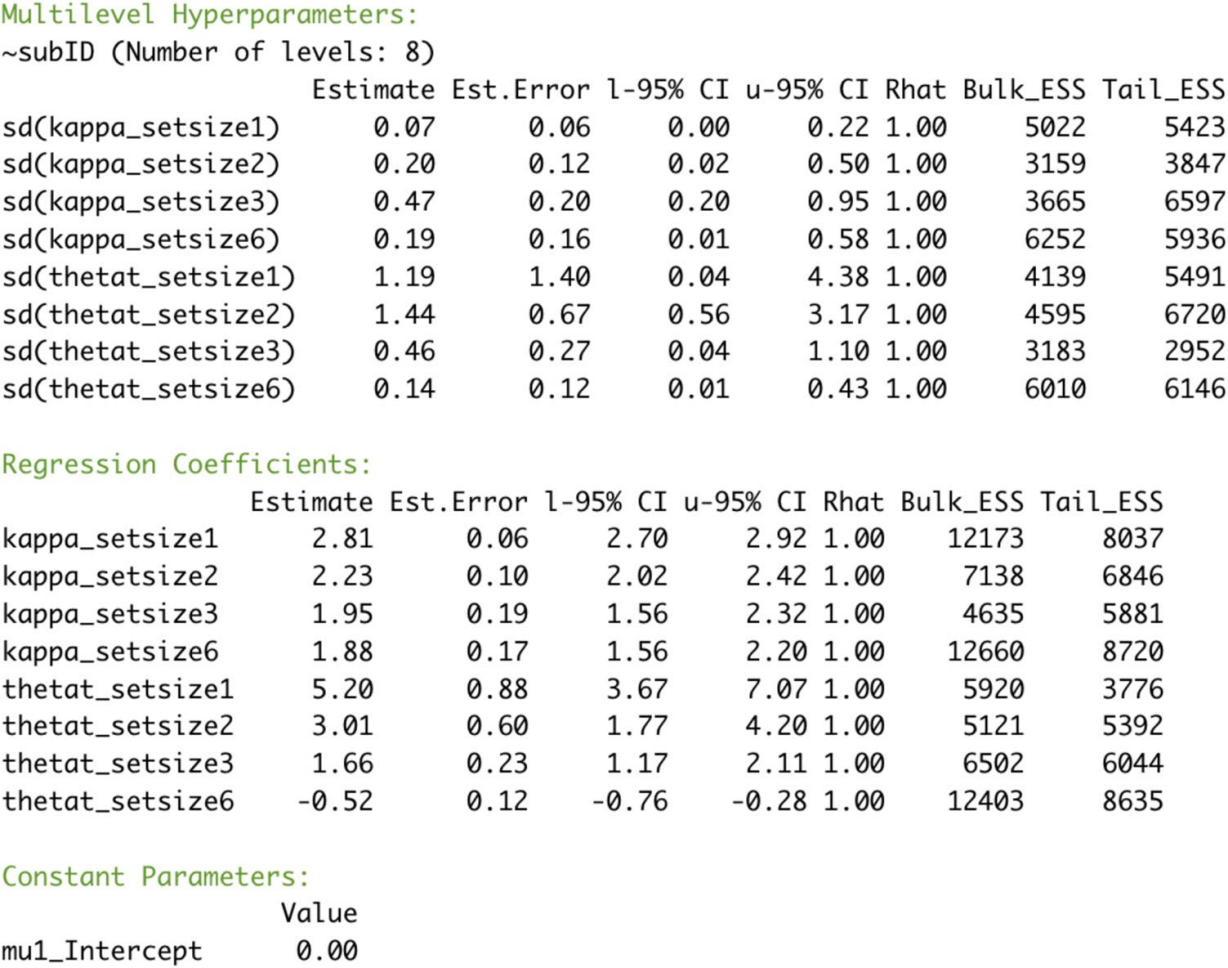
Summary output for the estimated parameters from the two-parameter mixture model of the Zhang & Luck (2008, Exp. 2) data estimated via *bmm*

**Model results** The general-purpose summary function will provide an overview of the estimated parameters of the model (see Fig. 6). The *Multilevel hyperparameters* section of the model summary (top section in Fig. 6) provides information on the group-level effects, in our example the variation of effects over subjects. Unless you are interested in individual differences, this section is not of primary interest. The main takeaway for our first example is that there is credible variation across all set sizes for both the precision of memory representations (*kappa*) and the probability for recalling an item from memory (*thetat*). The *Regression coefficients* section (bottom section in Fig. 6) summarizes the differences between parameters varying over the predictors we specified in our model formula, in this case set size. You need to keep in mind that the reported values are for the estimates on the parameter space (i.e., log-transformed kappa; mixing proportions instead of probabilities), so to interpret them more easily, you first need to transform them to their native scale. For more information about the output of summary, please consult the *bmm* manual.

There are several ways of extracting information from a fitted *bmm* object. For example, the *brms* functions fixef and ranef provide summaries of the estimated fixed and random effects.^11^ These can be used to transform both fixed and random effects from the parameter space to the native scale. In this case, we can use an exponential transformation to transform kappa estimates (i.e., $\text{exp}(kappa)$) and an inverse logit to transform theta estimates into probabilities. Additionally, we can convert kappa into the standard deviation of the von Mises with the k2sd function implemented in the *bmm* package. Keep in mind that this is scaled in radians because our response variable was provided in radians. Nevertheless, the standard deviation estimates in radians can also be transformed into standard deviations in degrees using ${sd}_{deg}=\frac{{sd}_{rad}}{\pi }*180$ or via the rad2deg function implemented in *bmm*. All these transformed estimates can then, for example, be used to plot fixed effects over conditions or evaluate the consistency of effects over subjects.

**Fig. 7:**
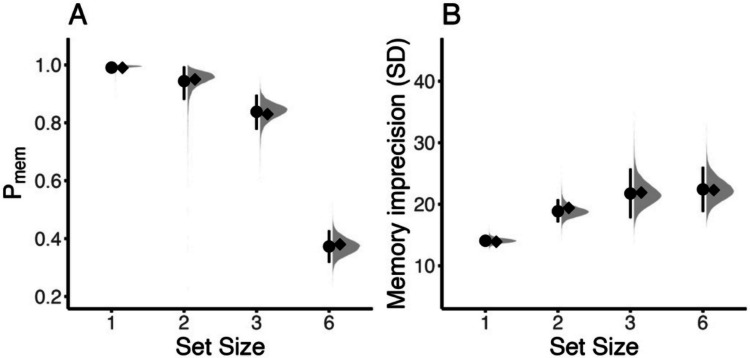
Replication of results from Zhang and Luck (2008) using the introduced hierarchical modeling framework for the two-parameter mixture model in *brms*. Panel **A** (on the left) shows the results for the probability of having an item in memory. Panel **B** (on the right) shows the results for the precision of memory representations. The posterior mean (point) and 95% credibility interval (line range) of the parameter estimates are shown in black. The average of the subject-wise estimates reported by Zhang and Luck (2008) are shown by the black diamonds. The gray distributions illustrate the whole posterior distribution of estimated parameters from the *brms* model implementation

Figure 7 shows the *bmm* parameter estimates for the Zhang and Luck (2008) data. Estimating the parameters from the two-parameter mixture model in *bmm* yielded a good model fit (see Fig. 5) and arrived at practically equivalent results to those reported in Zhang and Luck (2008). This demonstrates that our implementation works as intended and converges in parameter estimates with other estimation procedures. We will not go into detail for all the possibilities offered by *bmm* to evaluate model results. The R code in the online supplement illustrates some common steps in evaluating and plotting model results. Additional online material in several blogs or in the *bmm* and *brms* documentation provides ample introduction into post-processing of model results from *bmm* and *brms* models.

## Example 2: Estimating varying parameter values for a factorial design with within- and between-subject factors

In our second example, we used data reported in Loaiza and Souza (2018). In this experiment, a group of younger (*n* = 25) and older adults (*n* = 24) were instructed to memorize five colored disks distributed around an imaginary circle in the center of the screen. The retention interval was manipulated to be either short or long, and a variable number of cues (0, 1, 2) could indicate the item to be tested prior to recall (for details please refer to the original publication). The cues presented during the retention interval are thought to bring the item to be tested back into the focus of attention, thereby improving its accessibility and potentially the precision of the memory representation (Souza & Oberauer, 2016). We chose this example to illustrate how to adapt the implementation of the two-parameter mixture model for a more complex design combining between- and within-subject factors.

Except for the specification of the *bmmformula*, all steps are the same as in Example 1. We first set up the model object, passing the variable name of the response error “*dev_rad*” in the Loaiza and Souza (2018) dataset, which can be found in the GitHub repository of the tutorial but is not included in the *bmm* package:
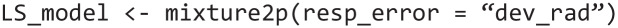


Then we only need to adapt our model formula to incorporate the respective independent variables to predict the mixture model parameters. For this dataset we have two within-subject factors (retention interval and cue condition) and one between-subject factor (age group). Here, we set up the model formula to estimate separate intercepts for the parameters in each age group and then estimate effects for the within-subject conditions by suppressing the estimation of an intercept using the 0 + coding:
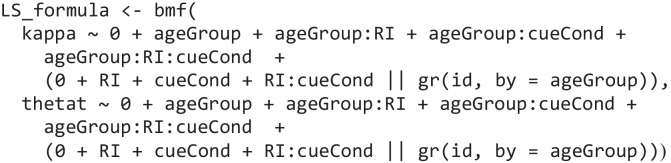


Specifically, this *bmmformula* first estimates separate intercepts for both the precision of memory responses (*kappa*) and the probability of recalling an item from memory (*thetat*) for the two age groups and then specifies main effects for the two experimental factors and their interaction for both age groups. We use this specification to compute evidence in favor of or against the effects of the experimental conditions on the two age groups more easily (see the section *Testing the hypothesis for parameter* estimates). Additionally, we estimate random effects reflecting individual differences in these estimates for the two within-subject factors. To avoid assuming that variability is equal between younger and older adults, we grouped the *id* variable over age group to allow for different degrees of variability across age groups (for details on these specific settings, please see the documentation of the *brmsformula* function and the vignette on the *bmmformula*).

**Fig. 8:**
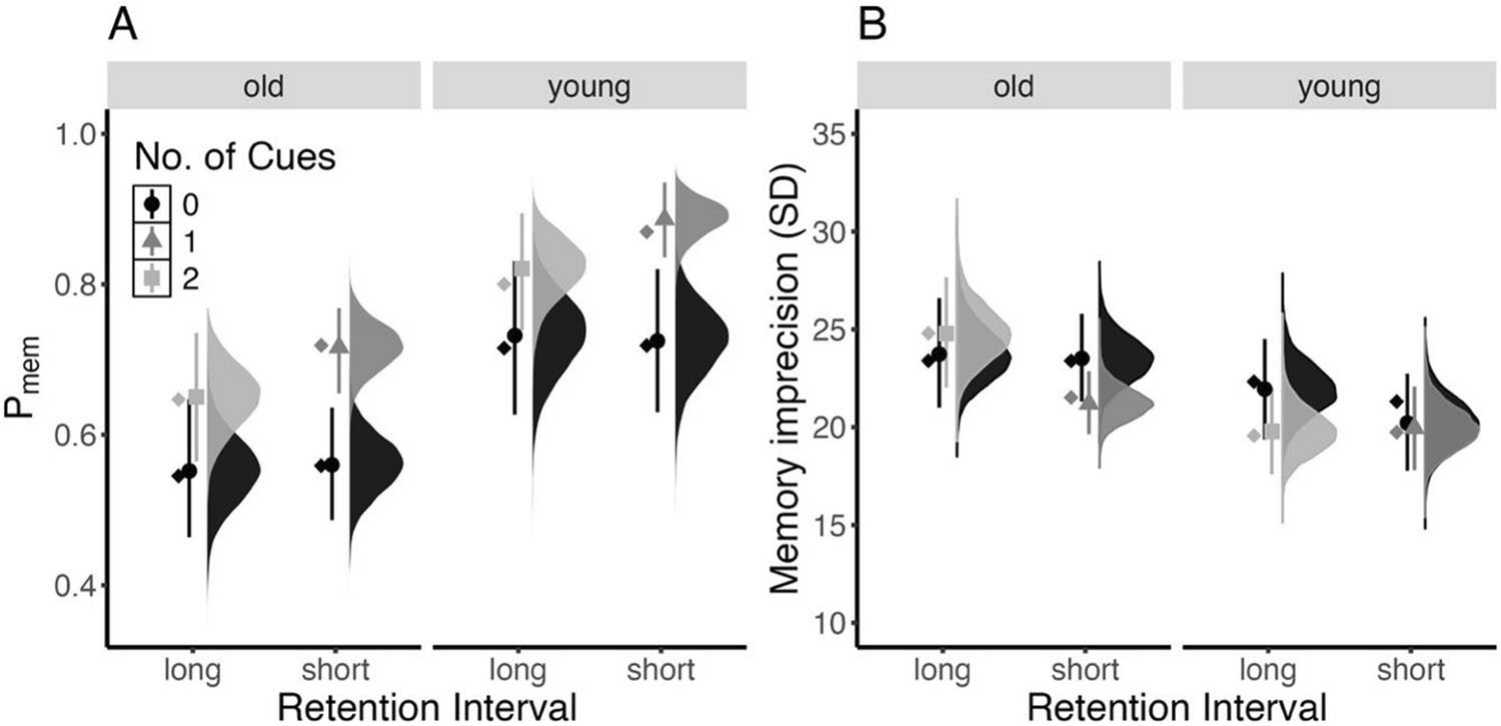
Reproduction of the results by Loaiza and Souza (2018) using the *bmm* implementation to estimate parameters from the two-parameter mixture model. The probability of recalling an item from memory (*p*_mem_) is shown in panel **A** (left side), and the imprecision of memory representation (*SD* of the von Mises) is shown in panel **B** (right side). The distributions shaded in black to light gray illustrate the whole posterior distribution of the respective estimates for the different numbers of cues. The dot indicates the posterior mean, and the line the 95% highest-density interval of posterior estimates. The diamond indicates the average estimate from the original publication

Using the specified model object and the adapted *bmmformula*, we estimate the parameters of the two-parameter mixture model by calling the *bmm* function:
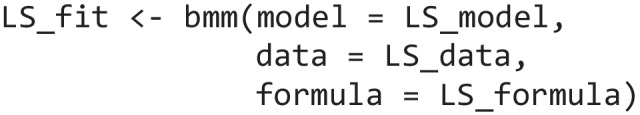


The posterior of the parameter estimates across the experimental conditions for both age groups are shown in Fig. 8.

## Testing the hypothesis for parameter estimates

There are several ways to evaluate hypotheses with Bayesian modeling techniques. Here, we will give a short illustration for testing a hypothesis using the estimated parameters from a *bmmodel*. For this, we can use the Savage-Dickey density ratio between the prior and posterior at a specified point of interest to quantify how much our belief has been updated by the data (Dickey & Lientz, 1970; Wagenmakers et al., 2010). This method can be used to approximate the Bayes factor comparing nested models that fix one parameter of a more complex model to a specific value, usually zero.

**Fig. 9:**
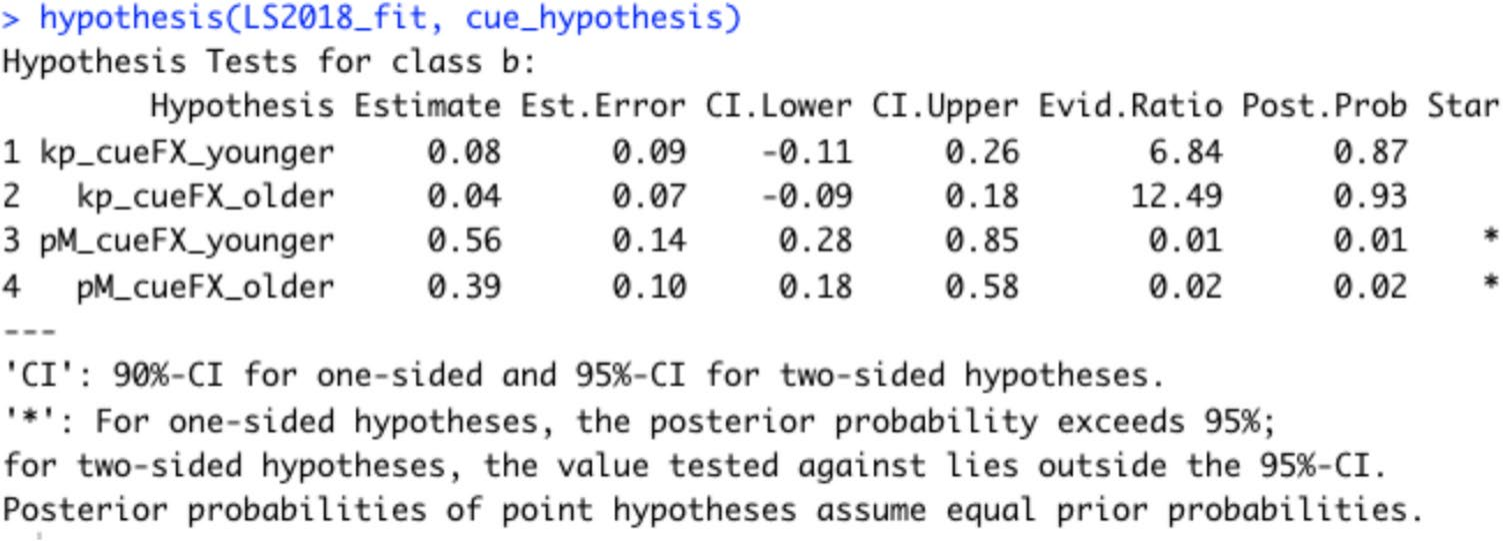
Output of the hypothesis function when testing whether there are effects of the retro cue on *kappa* and *thetat* for younger and older adults. The evidence ratio (Evid.Ratio) indicates evidence in favor of the specified hypothesis. In this case this reflects the BF_01_. To obtain the BF_10_, one can take 1/BF_01_. We recommend accessing the Evid.Ratio by storing the output of the hypothesis function in an object

A major benefit of *bmm* is its seamless integration with *brms*; thus, we can test hypotheses with this method on estimated parameters using the hypothesis function implemented in *brms*. To use this function, we first specify a set of hypotheses that we want to test. This is done by creating a character vector that contains named elements that define the comparisons of interest by referring to the parameters of interest via their names. For our example, we evaluate whether getting at least one cue improves the precision of memory responses and the probability of recalling an item from memory. Given the two age groups, we can do this separately for younger and older adults. Setting this up would look like this:
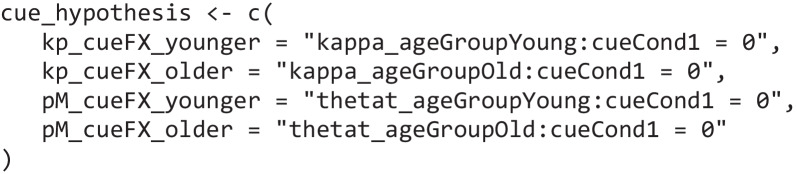


Then we can pass the specified hypothesis together with the *bmmfit* object to the hypothesis function and get posterior estimates of the specified comparisons. In addition, if we set the *sample_prior* option to TRUE when running the model with the bmm function, the output returns the Savage–Dickey density ratio (Evid.Ratio) in favor of the specified hypothesis, and posterior probabilities (Post.Prob) in support of the hypothesis (see Fig. 9).

As in the original publication, the probability of recalling an item from memory was higher with at least one retro cue compared to no cue for younger (BF_10_ = 94.97) and older adults (BF_10_ = 53.50), whereas precision of memory representation did not improve with retro cues (younger: BF_01_ = 6.84; older: BF_01_ = 12.49). Additionally, older adults had a lower probability of recalling items from memory than younger adults, BF_10_ = 3.01 × 10^6^, and lower precision of memory representations, BF_10_ = 5.29 (see the code in the online supplement for the calculation of these Bayes factors).

## Bayesian model comparison

A more general approach for drawing inferences when comparing Bayesian models is the use of Bayes factors that reflect the ratio of the marginal likelihood of different models. Notwithstanding the broader applicability of this method, there are also some downsides when using the method, namely (1) the need to estimate all models of interest, (2) the computationally intensive task of obtaining the marginal likelihood for each model, and (3) the need for setting appropriate priors, as these might affect the results. The first two points are mostly practical problems that will typically increase the time required for conducting the relevant model comparisons. The third point is more conceptual and is still discussed among Bayesians (Mikkola et al., 2023).

For example, if we wanted to test whether there is evidence for or against an interaction of the retro cue effect with the retention interval manipulation on both kappa and the probability of recalling an item from memory, we could compare the above-specified model including all main effects and interactions, with a reduced model that only estimates main effects of the retro cue and retention interval. For this we would simply drop the interaction term from the model formula:
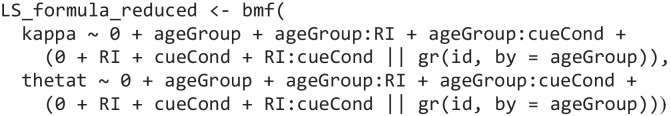


Recent simulations indicate that it is recommended to keep all random effects in the model, although the interaction is assumed to be zero (Oberauer, 2022); therefore, we have not dropped the RI:cueCond interaction from the random effects formula. We then fit this reduced model to the data by calling the *bmm* function and passing the model formula of the reduced model:
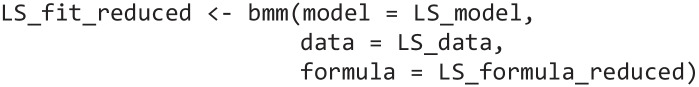


Having fit both the full and reduced model to the data, we can then obtain Bayes factors in favor of one model over the other by calling the bayes_factor function included in *brms*:
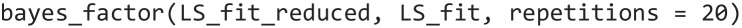


This function uses bridge sampling (Gronau et al., 2018) to approximate the marginal likelihood for both models. As this approximation of the marginal likelihood also relies on a sampling algorithm, we recommend increasing the number of posterior samples to at least 40,000 when estimating the model in order to obtain stable Bayes factor estimations. To gauge the variability of the Bayes factor estimation, you can also repeat the approximation of the marginal likelihood by setting the number of repetitions via the repetitions argument. 

For our example, we estimated the Bayes factor against an interaction of retention interval and retro cues 20 times, with both models having 12,000 posterior samples. Based on these estimations, we get a median BF = 9.59 in favor of the reduced model. However, over the 20 repetitions, the Bayes factors range from 3.89 to 63.04, indicating a broad range of moderate to strong evidence against the interaction of retention interval and retro cues in both age groups and for both kappa and the probability of recalling an item from memory. For more reliable inference, we would have to increase the number of posterior samples.

## Example 3: Estimating the three-parameter mixture model

In this example, we show how to estimate Bays et al.’s (2009) three-parameter mixture model using *bmm*. We apply the model to the data reported by Bays et al. (2009), which was a simple set size experiment akin to the one reported by Zhang and Luck (2008). Participants performed a continuous reproduction task where one, two, four, or six colors were presented simultaneously on the screen.

**Fig. 10:**
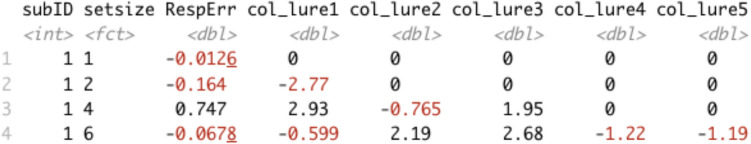
Example of data structure for the three-parameter model. One trial per set size is shown

First, we need to make sure that the data are in the correct format. The model expects that the outcome variable is response error relative to the target, and that the positions of the non-targets are also coded relative to the target. For example, if the target was a color with value 0.7, and the non-targets were 0.5, 0.9, and 1.1, they need to be re-coded by subtracting the target value, i.e., − 0.2, 0.2 and 0.4. Each non-target value should be stored in a separate column. If different set sizes are presented, there should be as many non-target columns as the maximum set size, i.e. 1, and for set sizes less than that, values in the extraneous columns should be coded as NA or zero. Figure 10 shows an example of a correctly prepared dataset. For an example of how to prepare data that contain the absolute responses and non-target values, see the vignette for fitting mixture models on the *bmm* website.

Again, we set up a model object so that *bmm* knows which variables to use for correctly specifying the model. For the three-parameter mixture model, we must provide more information than for the two-parameter mixture model. But setting up the model remains quite simple:
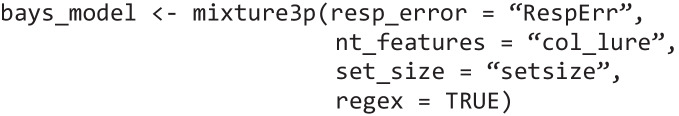


Again, we need to specify the response error (resp_error) relative to the target location. Additionally, we need to provide the variables containing the feature values of the non-targets relative to the target values (nt_features) to locate the distributions for swaps accordingly. Finally, we specify the set size variable, in particular when set size varies over trials. This is necessary to estimate the model simultaneously over several set sizes. Another useful feature when passing variable names is the regex option. This allows you to provide the general pattern that several variables, such as the nt_features, follow without having to manually paste together the variable names of all nt_features.

Then, just as in the first example, we specify the *bmmformula* for all parameters:
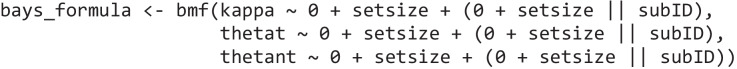


where *kappa* is the precision of the von Mises distributions, *thetat* is the mixing proportion for the target responses, and *thetant* is the mixing proportion for the non-target responses, *setsize* is a factor variable, and *subID* is the subject number. In this model, following Bays et al. (2009), we allow all parameters of the model to vary as a function of set size, and we also estimate a random slope for this effect for each subject.

**Fig. 11:**
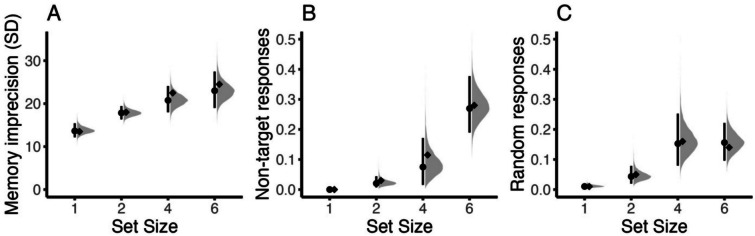
Reproduction of the results by Bays et al. (2009) using the *brms* implementation to estimate parameters from the three-parameter mixture model. **A** Imprecision of memory representation (*SD* of the von Mises). **B** Probability of non-target responses. **C** Probability of random responses (guessing). The distributions shaded in gray illustrate the whole posterior distribution of the respective estimates. The dot indicates the posterior median, and the line the 95% highest density interval of posterior estimates. The diamond indicates the average maximum likelihood estimate from the original publication

Then, we can estimate this model again using the *bmm* function:
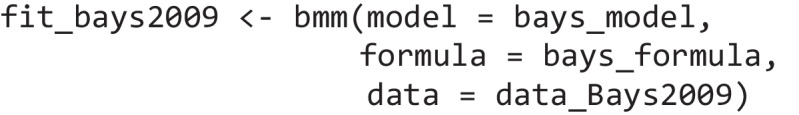


Figure 11 shows the estimates of the *bmm* model and the estimates originally reported by Bays et al. (2009). Despite some differences, all original parameter estimates lie within the 95% highest-density interval of the hierarchical model estimates, showing that the two estimation techniques converge.

## Example 4: Using priors to implement informative constraints and improve parameter estimation

**Fig. 12:**
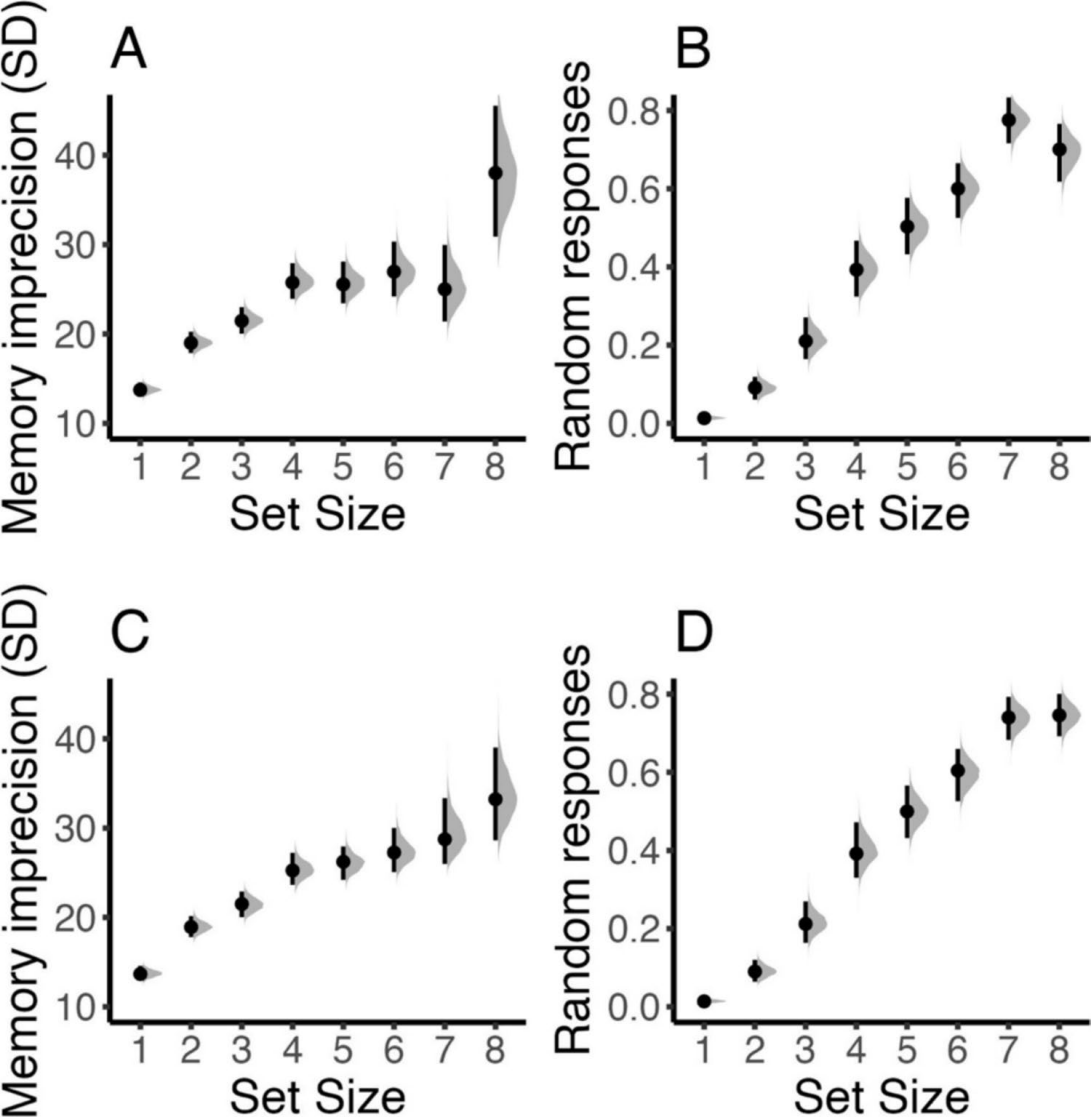
Parameter estimates of the unconstrained (**A** and **B**) and the monotonically constrained (**C** and **D**) two-parameter model in Example 4. **A** Imprecision of memory representation (*SD* of the von Mises). **B** Probability of random responses (guessing). The distributions shaded in gray illustrate the whole posterior distribution of the respective estimates. The dot indicates the posterior median, and the line the 95% highest-density interval of posterior estimates

For this example, we demonstrate how you can put prior constraints on parameters. We illustrate when this can be helpful using an unpublished dataset from our lab, in which people performed a delayed estimation task with set sizes from 1 to 8. We fit the two-parameter model as described in Example 1. Figure 12A and B show the posteriors of memory imprecision and probability of guessing from the two-parameter mixture model as a function of set size. While the general pattern is as expected, that is, worse performance with increasing set size, something unusual stands out for set sizes 7 and 8. The estimated guessing probability is higher for set size 7 than set size 8, but with higher memory precision. Based on existing literature, we know this pattern is highly unlikely. The problem is that when guessing probability is relatively high (*p*_guessing_ > 0.40), the mixture model can fail to recover parameters and can mistake high imprecision for guessing, leading to a trade-off in parameter estimates (Grange & Moore, 2022).

Following theoretical considerations and the existing literature (Oberauer & Lin, 2017; van den Berg et al., 2012), we expect memory imprecision and guessing probability to increase monotonically as a function of set size. Fortunately, the Bayesian framework allows us to set prior constraints on the estimates to force them to increase monotonically. This will likely provide enough information to the model to prevent a trade-off of imprecision with guessing. We chose this example because it occurred in our real work and represents a relatively complicated case, which will showcase the flexibility of the Bayesian estimation framework.

To enforce a monotonic increase of the parameters over set size, we need to do two things. First, we need to change the contrasts in the model, and second, we need to specify priors according to our theoretical assumptions over the parameters. By default, all regression models in R use dummy coding for factors. This means that the model estimates an intercept that corresponds to the parameter value for the first factor level (in this case, set size 1) and regression coefficients for each other level of the factor which correspond to the difference between each factor level and the intercept. This default coding can be extracted via the contrasts(data$set_size) command. In this case, this produces the following output:
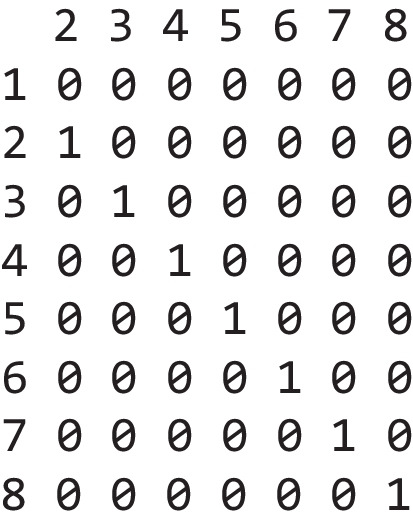


The row numbers reflect the eight different values of our factor (set size), and the columns reflect the desired contrast, in this case the default dummy coding. We want to set up a different type of contrast where each regression coefficient reflects the differences between the current factor level and the previous factor level. This would allow us to tell the model that this difference should always be positive or negative. Because the regression coefficients represent the differences between every pair of neighboring factors, this would enforce a monotonic increase over set size. This is not a tutorial on basic linear modeling, so without going into too much detail, the necessary contrast looks like this:
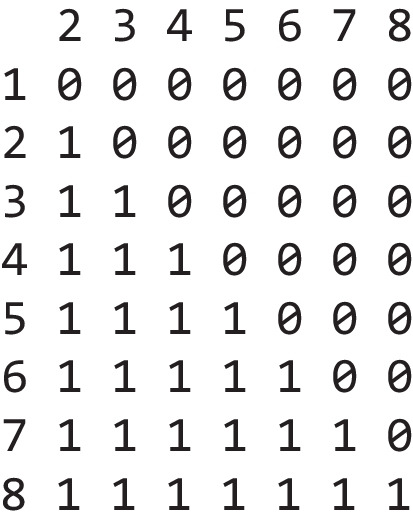


which we can specify with the following function:
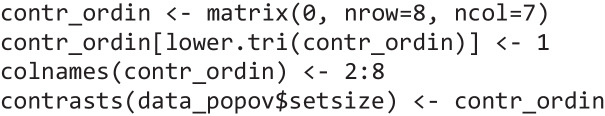


With these contrasts, any regression model fit to this dataset will estimate the difference between each two consecutive factor levels, instead of the difference relative to the intercept. We can set priors over these estimates to force them to be all non-negative, by setting the lower bound of the estimate to be 0 (lb = 0):
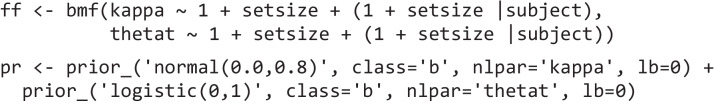


and then we estimate the model with.
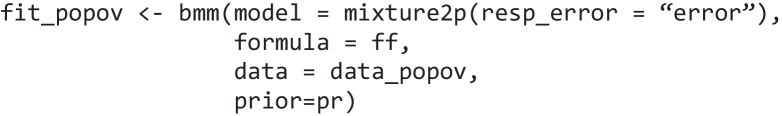


The new estimates are shown in Fig. 12C and D. In comparison with panels A and B, we can see that both the probability of guessing and the imprecision increase monotonically over set size, as we would expect based on prior theoretical and empirical work. Specifically, the model no longer estimates a dip in imprecision only at set size 7, together with more guesses relative to set size 8. It also produces a less inflated imprecision estimate for set size 8. It is important to note that imposing prior constraints can result in worse fit to the data. When comparing the model with and without prior constraints via Bayes factors, we found a median BF = 4.52 in favor of the model with monotonic constraints. However, over 20 repeated estimations of the marginal likelihood, for both models, the Bayes factors ranged from 2.37 × 10^−3^ to 154.30. Therefore, we cannot draw a strong conclusion whether imposing a monotonic effect of set size on both kappa and *p*_mem_ improved or deteriorated model fit based on the 30,000 posterior samples used for model estimation. This may also reflect that while the constraints have yielded parameter estimates in line with theoretical considerations that fit most of the data well, this might have deteriorated the fit to the data of single subjects. Given that the prior constraint we illustrate here is based on theoretical considerations about reasonable parameter estimates, this can still be reasonable for improving replicability. In the end, this dataset provides insufficient evidence for a non-monotonic effect of set size on kappa and *p*_mem_, and the effect would need to be replicated with new data or additional data would need to be collected.

This example illustrates a case when providing a somewhat informative prior to the model avoids prematurely accepting spurious effects by imposing theoretically motivated priors. From our perspective, the assumption that memory precision and probability of recall from memory decline with larger set sizes aligns with previous findings and theoretical assumptions (Oberauer & Lin, 2023; van den Berg et al., 2012; Zhang & Luck, 2008). Critically, the assumptions that these parameters increase monotonically includes that parameters of the model will no longer change between set sizes when the set size reaches a certain threshold (Pratte, 2020). Ultimately, the goal of this example was to illustrate the possibilities researchers have in considering informative priors to improve model estimation and for testing theoretical assumptions (Haaf & Rouder, 2018), and the validity of such assumptions and constraints obviously needs to be evaluated for each specific case. Providing a comprehensive collection of cases in which prior constraints might be reasonable is however beyond the scope of this tutorial.

## Example 5: Comparing parameter estimates from the three-parameter mixture model with the inference measurement model

For our last example, we will demonstrate how you can estimate parameters from the interference measurement model and illustrate what the interference measurement provides in terms of theoretical interpretation that goes beyond the three-parameter mixture model. For this, we have re-analyzed data from Experiment 1 reported in Oberauer et al. (2017). This experiment collected data from 20 young adults who had to do a continuous color reproduction task in which a variable number of color patches (from one up to eight) appeared on the screen, and then participants had to report the color of one of the patches on a color wheel. For each set size, 100 trials were collected.

**Fig. 13:**
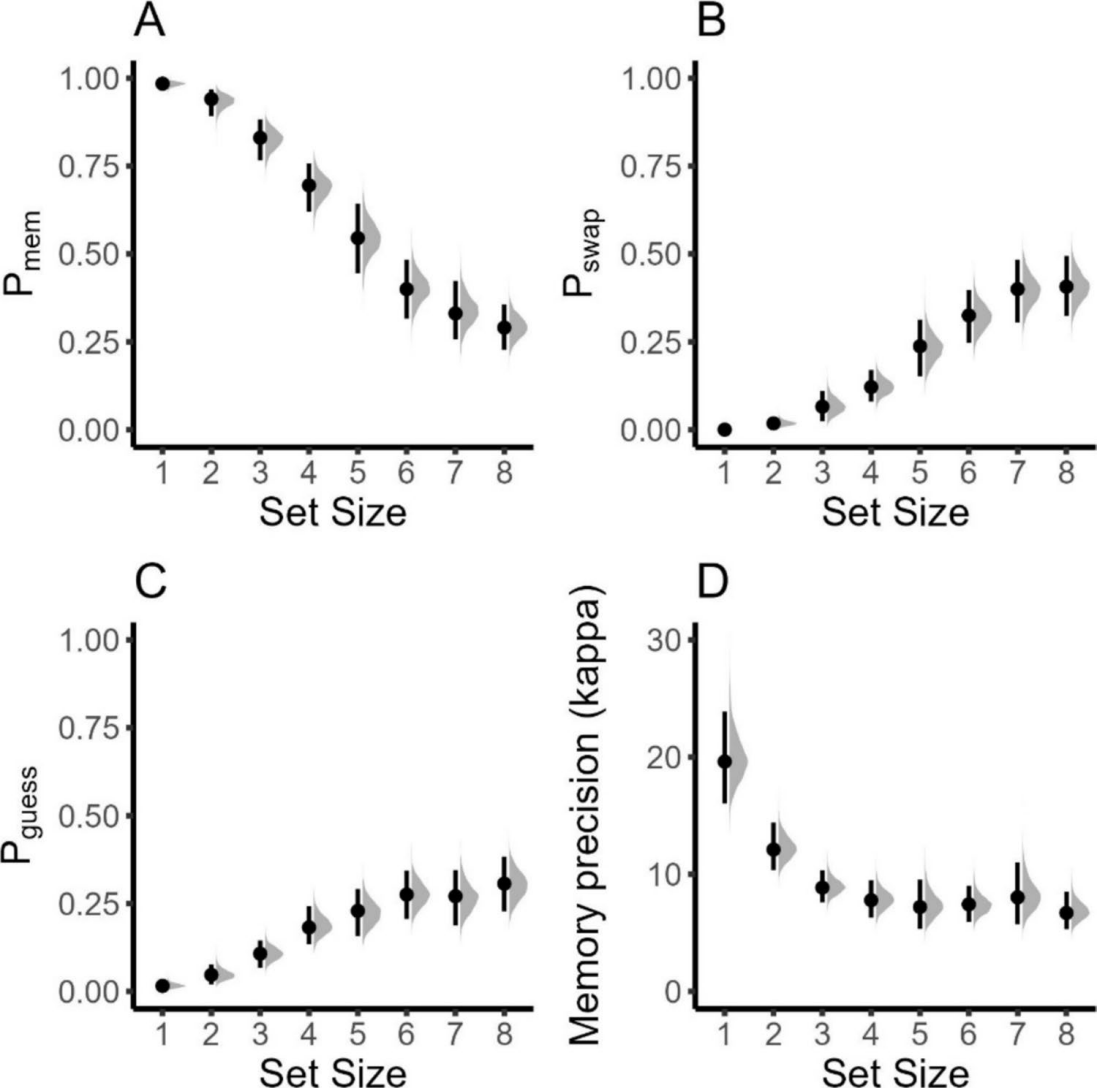
Estimates for the three-parameter mixture model for Experiment 1 reported by Oberauer et al. (2017). We freely estimated the probability of recalling an item from memory (panel **A**), the probability for swap errors (i.e., recalling a non-target, panel **B**), and the precision of memory representations (panel **D**). For completeness, we also show the probability for guessing responses (panel **C**). All plots show the posterior mean (dot) and the 95% highest-density interval (line range), as well as the full posteriors of the respective estimate (gray distribution) for all set sizes

First, we estimated parameters for the three-parameter mixture model from these data. Again, we start by setting up the model object:
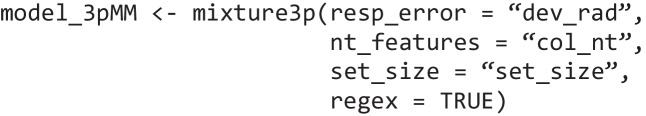


We then specify the *bmmformula* so that all three parameters vary over set size and pass it together with the model object and the data to the *bmm* function:
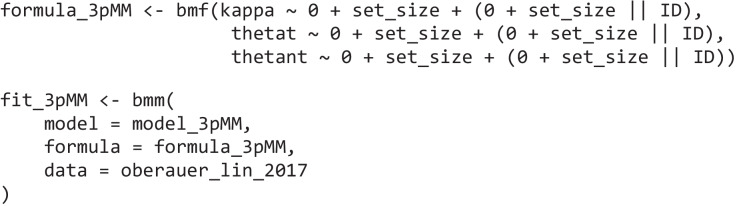


As for Example 3, we estimated the precision of memory responses (kappa) and both the proportion of target and non-target responses (*thetat* and *thetant*) for all set sizes. We also included random effects for all estimated parameters for all set sizes. The results for the parameters of the three-parameter mixture model are displayed in Fig. 13. Consistent with the results from previous examples, we see that the probability of recalling the target (*p*_mem_) from memory reduces from small to large set sizes (see Fig. 13A). Conversely, the probability of committing a swap error (*p*_swap_), that is, recalling one of the non-target items, increased from small to large set sizes (see Fig. 13B). Finally, the precision of memory responses (kappa) decreases with set size (see Fig. 13D). These results replicate the results reported by Oberauer et al., (2017; Fig. 20).

Neither the two-parameter nor the three-parameter mixture model provides a strong theoretical foundation of the processes underlying the mixture of different response distributions. The interference measurement model (IMM; Oberauer et al., 2017) attempts to fill this gap by assuming that the probability of recalling an item from one of the different mixture distributions is determined by several continuous sources of activation. Specifically, the IMM separates background noise *b* from general activation *a* for all items presented in the current trial, and context activation *c* for memory items that are associated with the context cued at retrieval. In the full IMM, it is additionally assumed that context activation can be generalized to non-targets following a generalization gradient *s* that reflects the precision of cue–target associations on the context dimension (for a more detailed description, please see Oberauer et al., 2017). However, as the estimation of the *s* parameter requires the additional information of spatial distance between target and non-targets, and more data to be estimated with sufficient precision, we will focus on the reduced IMM only including the *a*, *b*, and *c* parameter (i.e., the IMMabc).

Both the two-parameter and three-parameter mixture models are special cases of the full IMM (Oberauer et al., 2017). The IMMabc is mathematically equivalent to the three-parameter mixture model and provides a re-parameterization of the recall probabilities into different sources of activation. Additionally, discarding the general activation *a* from the IMM would result in a model equivalent to the two-parameter mixture model. The main benefit of the IMM over the two- and the three-parameter mixture models is that its parameters are grounded in an explanatory model of visual working memory (Oberauer & Lin, 2017) that provides parameters linked to specific activation sources in working memory.

Fitting the IMM to data in *bmm* is very similar to fitting the three-parameter or the two-parameter mixture model. First, we set up the model object. For the IMM, there is an additional version argument, to specify which version of the IMM should be set up. The default version is the full IMM. Here, we estimate the *abc* version and thus specify this accordingly:
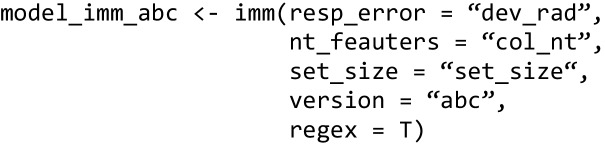


Then, we specify the *bmmformula* indicating which model parameters should be estimated depending on which experimental conditions:
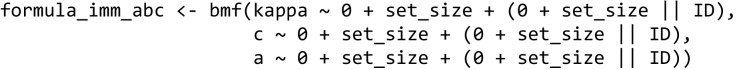


Again, we specify that the precision of memory responses *kappa* and both the context activation *c* and general activation *a* should be estimated for all set sizes. We again included random effects for all estimated parameters for all set sizes.

**Fig. 14:**
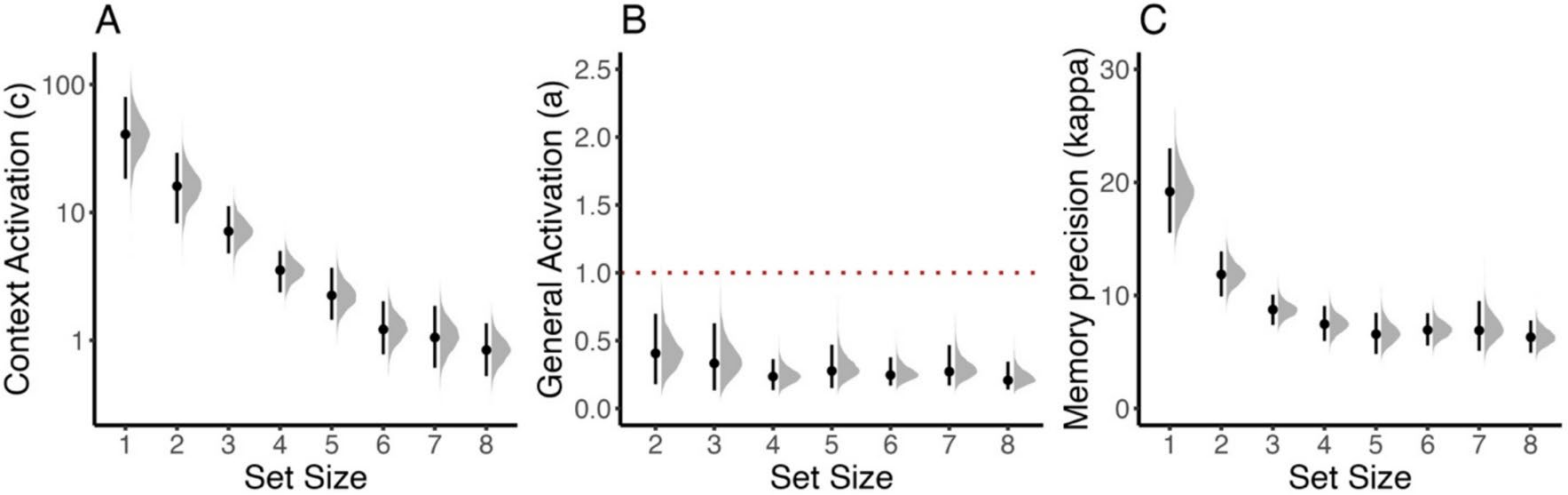
Parameter estimates for the IMMabc for Experiment 1 reported by Oberauer et al. (2017). We report the posterior estimates (means, 95% highest-density intervals, and the full posteriors) of the context activation (panel **A**) and the general activation (panel **B**). With respect to interpretation, these must be evaluated relative to the parameter fixed for scaling. In this case, we fixed the background noise (*n*) to 1 (illustrated by dotted red line in panel **B**). Panel **C** shows the estimates for the precision of memory representations. All parameters were allowed to vary between set size

Then, we can submit the model object and the *bmmformula* to the *bmm* function:
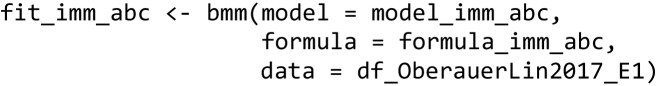


The resulting parameter estimates of the IMMabc are displayed in Fig. 14. Consistent with previous results, the context activation (see Fig. 14A), that is, the strength of the association between the color and the spatial location, decreases with larger set sizes (see estimates of the full IMM in Oberauer et al., 2017). The general activation however remains constant across set size, indicating that colors within each trial are held active at a similar activation level independent of set size. It is best to interpret the estimated activations relative to the activation component fixed for scaling. In this case, the background noise was fixed to 1. Thus, estimates below 1 for the general activation indicate that these activations were lower than the background noise (dotted red line in Fig. 14B), whereas context activation was higher than the background noise for most set sizes. Finally, as for the three-parameter mixture model, the precision of memory representations decreases with set size (panel C).

When comparing the results from the IMMabc to the three-parameter mixture model results, there are several things to note (for a model comparison via Bayes factors of the IMMabc and the three-parameter mixture model, see Appendix B): (1) The reduction in context activation qualitatively resembles the reduction in the *p*_*mem*_ parameter from the three-parameter mixture model. (2) Likewise, the estimates for the precision of memory representations are practically identical for the IMMabc and the three-parameter mixture model. However, (3) the pattern of general activation indicates no change (if anything, a reduction from small to large set sizes) whereas *p*_*swap*_ increases with larger set sizes. This indicates that the increase in swap errors is mainly due to the larger number of items a target can become confused with rather than each of the items having a higher activation. In addition, the reduction in context activation also reduced the difference in activation of the target relative to non-targets.

All in all, this example has illustrated that estimating the IMMabc is straightforward using the implementation in the *bmm* package. Currently, this implementation per default fixes the background noise to 1 and freely estimates all other IMM parameters. The *bmm* package has also implemented the two other versions of the IMM proposed by Oberauer et al. (2017).^12^ The “bsc” version assumes that swap errors occur only as a function of generalization on the context dimension and thus does not contain the general activation component but estimates the generalization gradient (*s*) instead. The “full” version combines confusions as a function of similarity on the context dimension, and confusions independent of similarity by estimating both the generalization gradient (*s*) and general activation (*a*). To estimate both these models, users must additionally provide the names for the variables coding the spatial distance between the target and all non-targets when setting up the model object. This is necessary for estimating the generalization gradient (*s*). An adapted model specification could look like this:
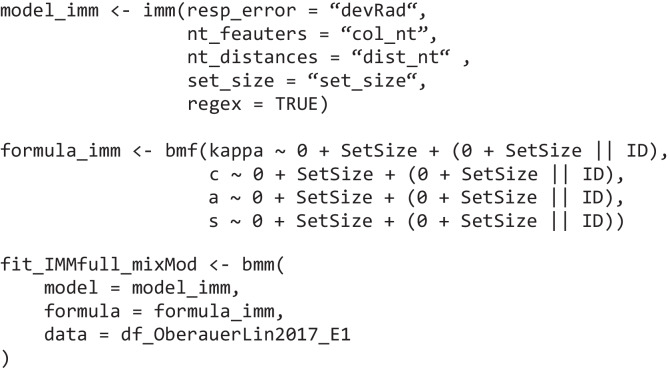


## Customizing and extending models implemented in *bmm*

The *bmm* package includes standard versions of the mixture models and the interference measurement model that are targeted for the continuous reproduction tasks often used in visual working memory research. However, sometimes researchers not only are interested in swap errors to other items presented in the same trial but also want to investigate proactive interference from previous trials or long-term memory representations (for an example, see Bartsch et al., 2024). In such cases, the proportion of swaps often needs to be dissociated into swap to items presented in the current trials and swap to items from previous trials or long-term memory. This is something the current implementations of the mixture models and interference measurement models in *bmm* do not entail.

For implementing such models, researchers have to customize the models implemented in *bmm*. A starting point to get an idea how that could be done is checking the *brms* code for all examples above. Specifically, the online supplement has code for the examples using the *bmm* package, and code for all examples using only *brms* that shows how the specification of these models would look without the *bmm* package. For brevity, we will not elaborate on all aspects of the *brms* implementations of these models. Instead, we want to point to some resources that provide support. As mentioned above, the manuscript by Bartsch et al. (2024) implements a mixture model including swaps to long-term memory representations based on the implementations we presented here and have implemented in the *bmm* package. All code for the analyses of this paper is shared and thus provides a good starting point for customizing these models for nonstandard use cases.

In addition, users can access all the information compiled by *bmm* that is then passed to *brms* (for a detailed introduction see the vignette on the *bmmformula*)*.* An easy way to achieve this is to extract information from a *bmmfit* object returned when estimating a model or creating a mock fit object for any of the *bmm* models you might want to customize. To do this, you can select “mock” as the backend and additionally specify the two arguments “mock” and “rename” when calling the *bmm* function:
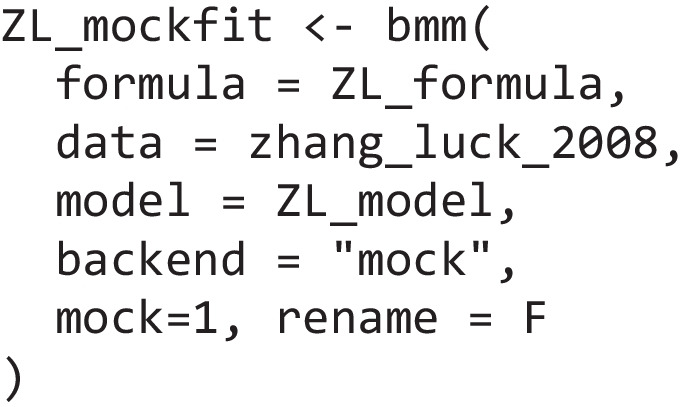


This will create an empty fit object including all information passed to *brms* but not containing any samples. The three necessary elements that you need to extract if you want to customize the models implemented in *bmm* are the distributional *family*, the *brmsformula*, and the data. All these can be accessed from the mock fit object: the *family* via ZL_mockfit$family, the *brmsformula* via ZL_mockfit$formula, and the *data* via ZL_mockfit$data. Detailed code illustrating which functions are used to set these up in the models we introduced here is provided in the online supplementary material for all examples. Essentially this code follows the five steps outlined in the section *Setting up a mixture model in brms*. You will see that setting up these models using only *brms* functions requires considerably more code, especially for the three-parameter mixture model and the interference measurement model. Finally, the webpage of *bmm* contains detailed Developer Notes that describe how models are set up in the package and what developers have to do to integrate new models into the package. To ease this process, the package also includes a “use_model_template” function that sets up all required files for a new model and provides guidelines on all things that need to be specified.

## General discussion

Measurement models of visual working memory have become a popular and useful tool to decompose raw behavioral performance in continuous reproduction tasks into separate meaningful model parameters (Oberauer et al., 2017). This tutorial described how researchers can use the new *bmm* package to estimate the two-parameter mixture model (Zhang & Luck, 2008), the three-parameter mixture model (Bays et al., 2009), and the interference measurement model (Oberauer et al., 2017) in a hierarchical Bayesian framework. The *bmm* package builds on *brms*—arguably one of the most popular and powerful R packages for Bayesian regression modeling—and aims to makes it as easy and accessible to specify and estimate these models in a Bayesian hierarchical framework. Given the close integration with *brms*, users can use much of the functionality integrated in *brms* for evaluating the models in *bmm*. Additionally, we provide a GitHub repository with well-documented code for each of the five examples using both *bmm* and *brms* to implement the different measurement models, which can be a useful learning tool. The five examples we presented demonstrate the flexibility of these implementations. Any model that can be specified with the *bmmformula* syntax can be estimated—including single and multifactorial designs, within- and between-subject designs, continuous and categorical predictors, and various random effect structures. In contrast to the typical two-step maximum likelihood procedure, researchers can predict different model parameters as a function of different conditions, rather than allowing all parameters to vary across all conditions.

When estimating mixture models with *bmm* or *brms*, users should keep several important things in mind:

- All responses and item values should be coded in radians, not degrees.
- The response variable should contain the response error, i.e., the response relative to the target.
- If estimating the three-parameter model or the interference measurement model, you need to provide the values of the non-target items, relative to the target item. For the “bsc” and “full” version of the IMM, you need to additionally provide the spatial distance of non-targets to the target.
- For efficient estimation, *brms* transforms the *kappa* and probability parameters (as we explained in the section *Explanation of model parameters and specifying the model formula*). The output of the estimated model (via the *summary()* function) presents these transformed parameters. Thus, you need to reverse the transformation by exponentiating *kappa* values and putting *thetat* and *thetant* through the softmax transformation in order to obtain the probabilities associated with different distributions (see the examples on GitHub for some post-processing routines).
- Before interpreting the estimated parameters, it is important to check convergence of the MCMC sampling and examine the overall fit of the model (e.g., by visual inspection of the predicted versus the observed data).
- For statistical inference, you could either use the highest-density intervals of the parameter estimates or, alternatively, obtain Bayes factors by comparing posterior probabilities with prior probabilities (Wagenmakers et al., 2010) for specific hypotheses or by comparing models with and without the predictor of interest (Gronau et al., 2018; Kruschke, 2011; Rouder et al., 2012).

## Implications for individual differences research

An important aspect of using cognitive measurement models is obtaining process-level indicators of individual differences and integrating them into correlational analyses (Frischkorn & Schubert, 2018; Frischkorn et al., 2022a, 2022b). The inclusion of random effects naturally integrates the estimation of individual differences as random effects over subjects, just the way we implemented them in all examples above. If researchers are interested in which parameters show credible individual differences, they could simply compare a model including random effects over subjects for a parameter of interest to a model not including the random effects. This is actually something that has been recommended to avoid biases in inferences about experimental effects in cases of a mis-specified random effects structure (Oberauer, 2022).

The benefit and challenge in using Bayesian estimation techniques for individual difference research is that unlike maximum likelihood estimation techniques, they do not return a single estimate for subject-level parameters, but a full posterior distribution. On the one hand this provides a more adequate representation of uncertainty in subject-level parameter estimates; on the other hand, it requires some additional consideration in the subsequent use of subject-level parameter estimates in correlational analyses. In addition, the inclusion of shrinkage can result in a suboptimal fit of estimated model parameters to data of a single subject. Although this might seem less than ideal, the shrinkage included in hierarchical modeling frameworks reduces estimation error and improves the recovery of subject-level parameters, although it occasionally might not fit the data of each individual subject perfectly (see the Appendix for a comparison of hierarchical versus subject-wise parameter recovery). In fact, hierarchical modeling techniques oftentimes can yield good parameter recovery with less data per subject (Vandekerckhove et al., 2011).

With respect to the question of how to deal with the full posterior distribution for subject-level estimates, there are two options that adequately deal with the uncertainty of subject-level parameter estimates:

1. Users can include covariates directly into the *bmmformula* and predict variations in model parameters by external covariates just like in any other hierarchical regression model. It is important to note that the regression weights reflect unstandardized regression weights that depend on the estimated standard deviation over subjects for the respective model parameter (see Frischkorn, von Bastian, et al., 2022a; Frischkorn & Oberauer, 2024).
2. Users can draw a set of random samples (typically 100 up to 1000) from the posterior of each subject-level parameter and enter them into traditional covariance analysis techniques (e.g., correlation, regression, or structural equation modeling), repeating the model estimation with each set of random samples of the subject estimates. This will provide an adequate approximation of the posterior of the correlation to external covariates (for an example, see Frischkorn & Oberauer, 2024; Rey-Mermet et al., 2021).

For a quick analysis of relations to external covariates, users can also extract a measure of central tendency for each subject-level parameter (e.g., the posterior mean or median) and use these in correlational analyses. However, this again reflects a two-step analysis approach that will underestimate the standard error of regression weights or correlation coefficients and thus lead to inflated alpha-error rates. Nevertheless, the estimated effect size is typically not affected by this two-step approach. Thus, this approach can serve as an adequate approach for approximating the size of relationships, but not their credibility.

In sum, the implementations of cognitive measurement models presented here provide excellent estimation tools for individual differences research. Users can extract subject-level parameter estimates from all models including random effects over subjects and propagate the uncertainty in subject-level parameter estimates through different analysis techniques by sampling from the posterior or directly including covariates in the model. Furthermore, the integration of structural equation models in *brms* is currently underway so that a combined multilevel–structural equation modeling approach for individual differences might be available for the cognitive measurement models implemented here as soon as this is integrated in *brms*.

## Theoretical considerations

Computational models of memory and cognition are often split into two classes: measurement models and process or explanatory models. Both types of models decompose behavior into separate meaningful parameters, but in contrast to process models, measurement models typically do not provide mechanistic explanations for differences in experimental conditions. Instead, measurement models allow their parameters to vary across experimental conditions to account for these differences. Nonetheless, measurement models provide considerable benefits (Farrell & Lewandowsky, 2018; Frischkorn & Schubert, 2018; Frischkorn et al., 2022a, 2022b; Oberauer et al., 2017). They decompose the observed behavior into meaningful parameters and provide a theoretically grounded interpretation of these parameters. Thus, measurement models enable researchers to evaluate experimental effects on the level of these parameters instead of the behavioral responses, providing a more fine-grained perspective with respect to the potential inference.

In particular, the interference measurement model is linked to a more complex explanatory model of visual working memory (Oberauer & Lin, 2017, 2023) and thus inherits some of the mechanistic processes implemented in this model. Therefore, its parameters can be interpreted in terms of theoretical processes or activation sources for which the contribution to observed behavior is clearly specified. The fact that both the two- and three-parameter mixture model are mathematically equivalent to special cases of the interference measurement model additionally highlights that these models themselves do not provide evidence in favor of either slot (Adam et al., 2017; Ngiam et al., 2022; Pratte, 2020; Zhang & Luck, 2008) or resource accounts (S. Ma et al., 2022; W. J. Ma et al., 2014; van den Berg et al., 2014) of working memory. Therefore, in fitting a two-parameter mixture model, you should not assume that visual working memory is limited by slots. To do so, additional assumptions would have to be added to these models (e.g., memory precision following a specific function across set sizes) to account for theoretical ideas put forth by slot or resource accounts of visual working memory. Additionally, the fact that the IMM assumes continuous activations underlying the retrieval of memory representations adds a third account to the explanation of capacity limits in visual working memory, the binding hypothesis that assumes that capacity limits a person’s ability to form and maintain bindings (Oberauer, 2021).

Although the different measurement models introduced in this paper are linked to different theoretical perspectives, the goal of this paper is not to discuss and compare these theoretical models of working memory. Therefore, we deliberately did not weigh in on the discussion regarding which of the different measurement models is considered superior and better supported by empirical data, except for illustration purposes, and refrained from an in-depth explanation and discussion of theoretical advantages and disadvantages of these models. Instead, we pointed to relevant resources where necessary.

The goal of this tutorial was to introduce the implementation of three measurement models for continuous reproduction tasks in *brms* and introduce the *bmm* package that aims to facilitate the use and application of these measurement models. We hope that the implementations we introduced here will enable more researchers to compare these different models and contribute towards the evaluation of benefits and problems of these different measurement models. Generally, we think that analyzing data on the level of cognitive processes will provide more refined insights into the effects of different experimental manipulations and advance our field towards a more comprehensive explanation of working memory and its limited capacity.

## Towards a broad range of easy-to-use cognitive measurement models

This tutorial is deliberately limited to mixture models that provide measurement models for continuous reproduction tasks. Obviously, research on visual working memory and working memory more generally uses a broad range of different tasks, procedures, and materials, such as change detection paradigms, and digits, letters, or words as memoranda. Although it is reasonable to assume that the distinction between recall from memory, random guessing, and variable precision or strength of memory representations should be relevant across a broad range of tasks (Oberauer & Lewandowsky, 2019; Oberauer & Lin, 2023), modeling behavioral responses in change detection tasks (Lin & Oberauer, 2022; Robinson et al., 2023) or tasks using discrete stimulus material requires entirely different implementations of these concepts (see, for example, Oberauer & Lewandowsky, 2019).^13^ Similarly, other recently proposed measurement models for continuous reproduction tasks, such as the target competition confusability model (TCC; Schurgin et al., 2020) and the signal discrimination model (SDM; Oberauer, 2021), do not use a continuous distribution, such as the von Mises distribution, to model the recall error in continuous reproduction tasks and instead introduce several additional steps and different distributional assumptions.^14^ Therefore, we felt that to keep this tutorial accessible and limited in length, it was best to focus only on a set of models that use similar distributional assumptions.

**Fig. 15:**
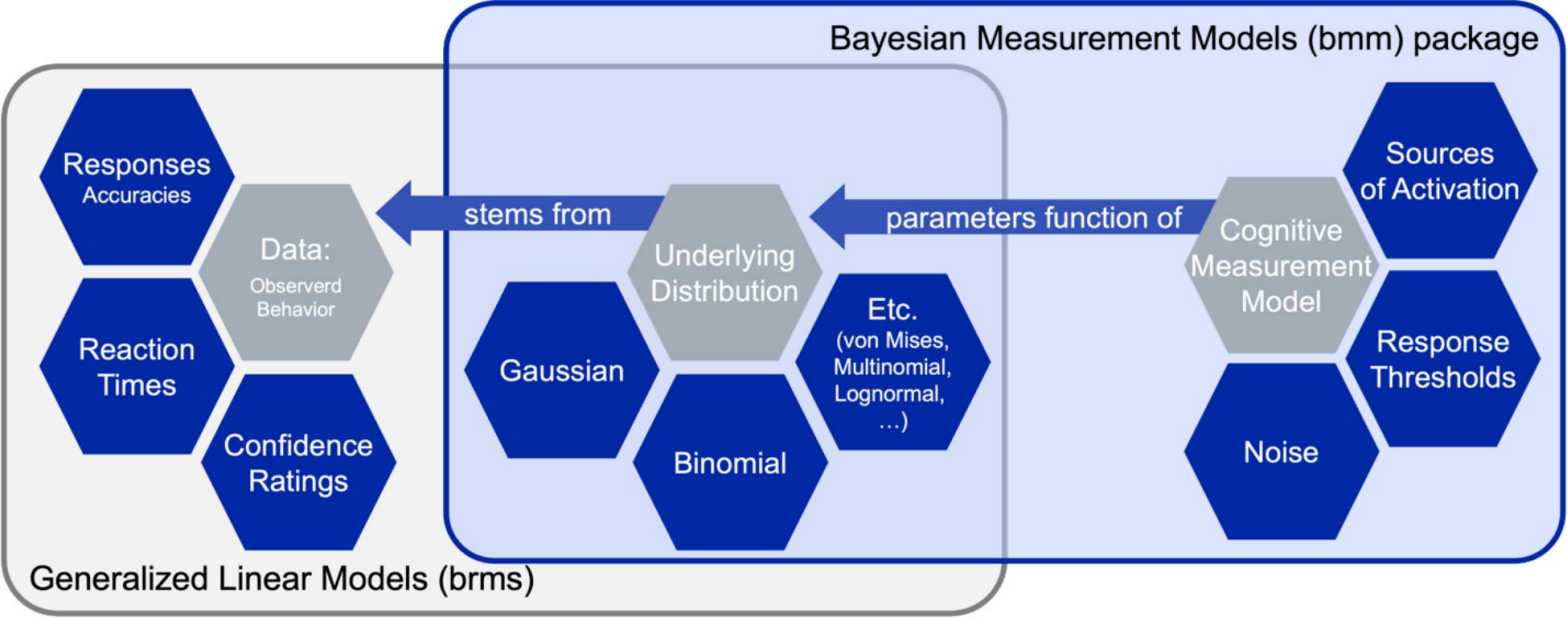
Illustration of the relationship between generalized linear models to cognitive measurement models. Generalized linear models provide a distributional description of the observed data. For example, accuracy data that can stem from a binomial process with a certain number of trials and probability of success, or reaction time data can stem from a lognormal distribution with a certain mean and standard deviation. Cognitive measurement models provide an additional decomposition of the distributional parameters, that is probabilities of success, means, or standard deviations, into cognitive processes assumed to underlie the observed behavior. This often integrates several distributional parameters or additional information about the experimental setup or stimuli

Nonetheless, the setup of the *bmm* package provides the foundation for the implementation of a broad range of cognitive measurement models, while still retaining the accessible and easy-to-use features that we highlighted in this tutorial. On a more abstract level, the implementations for the measurement models we presented here illustrate that cognitive measurement models can be specified as an extension of distributional models of observed data (see Fig. 15 for an illustration). Previous research has already highlighted the benefits of more closely aligning the modeling of behavioral data with distributions that more adequately resemble core features of the observed data (Haines et al., 2020). For example, instead of modeling aggregated reaction times with standard Gaussian models, using generalized linear mixed models assuming the data to follow a lognormal or inverse Gaussian distribution dramatically improves the inference and strengthens the conclusion that can be drawn from the analyses. The core insight of the implementations presented here is that many cognitive measurement models can often be specified as distributional models for which the distributional parameters of the generalized linear mixed model are a function of cognitive measurement model parameters (again see Fig. 15 for an illustration). These functions that translate the cognitive measurement model parameters into distributional parameters are what we essentially implemented for the three measurement models discussed in this tutorial.

**Fig. 16:**
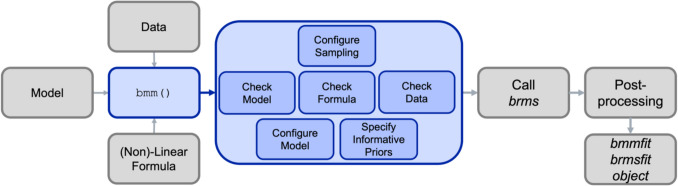
Flowchart for the functionality of the bmm function. The user only needs to pass their data and specify the model and formula in the bmm function; the bmm package then takes care of some checks, configuring the model and specifying reasonable default priors, and then passes the compiled information to brms

To avoid the need for researchers to code the translation functions of cognitive model parameters into distributional parameters themselves each time they want to use a specific model, we developed the *bmm* package. Additionally, the *bmm* package also performs some additional checks for the data provided with the model, specifies reasonable priors for the different model parameters, and ensures that model estimation runs as efficiently as possible. In this way, data can be analyzed on the level of latent cognitive processes instead of observed behavior. For this, you only have to specify the linear model formula predicting which of the cognitive model parameters vary as a function of experimental manipulation and select the appropriate model to be fit in the *bmm* function (see Fig. 16).

As of now, the *bmm* package allows fitting only a limited set of cognitive measurement models. The version released on CRAN includes the three mixture models introduced in this tutorial, as well as the signal discrimination model (Oberauer, 2021). The current development version that is available via GitHub also includes the memory measurement model (Oberauer & Lewandowsky, 2019) for categorical responses. Prospectively, we plan to extend the *bmm* package with implementations of measurement models for a broader range of tasks, such as signal detection models (DeCarlo, 1998; Vuorre, 2017), additional recent models for continuous reproduction tasks (e.g., the TCC; Schurgin et al., 2020), and models for reaction time data (Annis et al., 2017; Peña & Vandekerckhove, 2024), while still retaining the easy and accessible usability we presented here. But given the additional coding work required for these implementations, the time it would take to explore them, and an in-depth introduction on how to use these models, this was beyond the scope of this tutorial. Instead, this tutorial constitutes the first step in the development of a more general and broadly applicable package aiming to facilitate the use and application of cognitive measurement models in psychology in general.

## Conclusion

We have demonstrated how to implement and estimate three different kinds of mixture models for visual working memory tasks in a newly developed R package for Bayesian measurement models, *bmm*. We also elaborated how these implementations can be performed using the R package *brms* without relying on any additional functions from the *bmm* packag*e*. To enable a better understanding of our examples, we share R code for both the implementation in *brms* without relying on *bmm* functions and the implementation using the *bmm* function in a GitHub repository. We hope that these implementations will enable more researchers to fit hierarchical Bayesian mixture models, and in particular the interference measurement model, to visual working memory tasks with continuous reproduction recall. We are curious to see how the benefits of the current implementation—for example, the possibility to predict parameters of the implemented models by continuous predictors (e.g., confidence ratings or ratings of task focus in mind-wandering research)—will aid researchers in gaining insight into questions that could not be addressed with other implementations of these measurement models so far.

## Supplementary Information

Below is the link to the electronic supplementary material.

Supplementary file1 (DOCX 2377 KB)

## Data Availability

All data used in this tutorial are openly available on GitHub: https://github.com/GidonFrischkorn/Tutorial-MixtureModel-VWM/tree/main/scripts/

## Footnotes

1 For example, a Google Scholar query for “‘mixture model’ AND ‘visual working memory’” returns 930 results; the article by Zhang and Luck (2008) which introduced mixture modeling in visual working memory has been cited 1677 times; the MATLAB package *MemToolbox* which implements various mixture models for visual working memory has been cited 252 times (Suchow et al., 2013).

2 In addition to the measurement models we present and discuss here, there are other, more recent measurement models such as the target competition confusability model (TCC; Schurgin et al., 2020) or the signal discrimination model (SDM; Oberauer, 2021). However, these models differ in their underlying assumptions and are formally distinct from the models we discuss here. Therefore, we decided against including these models in this tutorial. The discussion includes a more detailed description of their differences.

3 While such biases might be negligible with sufficient data, a clear threshold of what constitutes “sufficient” data depends on the measurement model. The hierarchical Bayesian approach using one-step inference eliminates such biases even with little data (Boehm et al., 2018; and see Appendix A); from a conservative perspective, this approach is therefore to be preferred. Nevertheless, there are examples in which subject-wise ML and hierarchical Bayesian estimation yield similar results (e.g., Xie et al., 2023). However, Bayesian methods still have advantages over frequentist analysis when accepting the null hypothesis, as they can indicate when evidence is insufficient to accept either the null or alternative hypothesis. This can also be seen in the results from Xie et al. (2023), where some *p*-values are far from significant, *p* > .40, but Bayes factors in favor of the null indicate inconclusive evidence.

4 Scripts for all examples are available on GitHub. in the *scripts* folder.

5 You can compare the model specification for Example 3 in *brms* versus *bmm* via the *Example3_Bayes2009.R* file in *scripts/brms_examples* and *scripts/bmm_examples.* In *bmm*, users only specify a formula for the model parameters, and the package then specifies all additional information required by *brms*. Thus, the relationship between *bmm* and *brms* is the same as that between brms and Stan: *brms* handles model specification, data coding, and other labor-intensive procedures, and then generates the relevant Stan code. For measurement models, *bmm* takes care of similar tasks that become tedious and complex in *brms*, before submitting the model to *brms* for estimation.

6 *bmm* and its installation instructions are available on GitHub.

7 In principle, the mixture function in *brms* can be used to specify any finite mixture of the supported data distributions. This is a powerful feature that generalizes to any case in which the data do not follow a single distribution, and we hope that this tutorial enables researchers outside visual working memory research to consider mixture models in cases where they might provide additional theoretical insight. One important limitation of the implementation of mixture families to date is that the number of distributions cannot be determined or estimated by the data and thus must be equal for all conditions. This requires additional variables and more complex model code for a general-purpose implementation of the more complex measurement models for visual working memory tasks. This will be explained when these models are introduced.

8 *mu1* and *mu2* are fixed to 0, because the mean of the error and the uniform distributions are 0, *kappa2* will be fixed to 0 because the second von Mises distribution should be uniformly distributed, and theta2 will be fixed to 0 to identify the softmax transformation.

9 This is the value used for generating the guessing distribution in Fig. 2.

10 It is possible to estimate the location of the target distribution in *bmm*. Details on freely estimating internally fixed parameters can be found in the vignette on the *bmmformula*.

11 Please note that random effects are centered, meaning that parameter estimates reflect deviations from the respective mean, not the absolute estimate for each subject. Therefore, you need to add the fixed effect for the respective condition to the random effects and then transform them to the absolute scale. Otherwise, you will obtain incorrect estimates for the different subjects.

12 Please see the “scripts/bmm_examples” folder on the GitHub repository for examples implementing both the IMMbsc and the IMMfull for Experiment 1 reported by Oberauer et al. (2017).

13 First, the underlying response distribution for these tasks is different: In change detection tasks, responses follow a binomial distribution, whereas with discrete stimulus material, responses follow a multinomial distribution of different responses or response categories. Second, these different response distributions necessitate a different translation of the assumed cognitive states a person might be in, into the observed responses (Lin & Oberauer, 2022). Due to these complications, we restricted ourselves to mixture models for continuous reproduction tasks that all share the von Mises distribution as response distribution and assume that behavioral responses represent a mixture of different von Mises distributions.

14 Specifically, they assume a continuous activation function (either a Laplace or a von Mises distribution) for the responses around the circle and model recall as a competitive selection of all 360 responses possible on the color wheel. Formally, this resembles a multinomial response distribution for which the probability of selecting the different response options is derived as a function of the continuous activation function. The dependencies in the probabilities of recalling neighboring response options is introduced by the continuous activation function but not included in the response scale being continuous.

## Code availability

All code used in this tutorial is openly available on GitHub: https://github.com/GidonFrischkorn/Tutorial-MixtureModel-VWM/tree/main/scripts/).

## Declarations

### Open practices statement

All code and data used in this tutorial are openly available on GitHub: https://github.com/GidonFrischkorn/Tutorial-MixtureModel-VWM/tree/main/scripts/. The *bmm* package can be installed from GitHub as well: https://github.com/venpopov/bmm. The output for the models presented here is available on OSF: https://osf.io/vsrz4/
