# Supplementary material for: A tutorial for estimating Bayesian hierarchical mixture models for visual working memory tasks: Introducing the Bayesian Measurement Modeling (bmm) package for R
Source: Behav Res Methods. 2025 Apr 14;57(5):144. doi: 10.3758/s13428-025-02643-0 (PMC11996974; doi:10.3758/s13428-025-02643-0)
Supplement: Supplementary file 1 — Supplementary file1 (DOCX 2377 KB) [file 13428_2025_2643_MOESM1_ESM.docx]

**Appendix A: ML vs. BMM parameter recovery of the two-parameter mixture model**

**Procedure and Design**

First, we generated subject parameters for 20, 40, or 80 subjects of the two-parameter mixture model. Second, using these subject parameters we generated data using the random generation function for the two-parameter mixture model implemented in bmm (rmixture2p()) with four different numbers of observations per subject: 25, 50, 100, and 200. Third, we then estimated parameters for the two-parameter mixture model using subject-wise maximum likelihood estimation implemented in the mixtur package, and using hierarchical Bayesian estimation implemented in the bmm package. We repeated this parameter recovery procedure 200 times for each condition combination (i.e. a total of 2400 = 3 * 4 * 200 times) to approximate the variability in parameter estimation due to random noise in the data generating process.

Over repetitions, the means of $P_{mem}$ & $\kappa$ for generating subject parameters were randomly drawn from uniform distributions. For $P_{mem}$ means ranged from 0.3 to 0.95, and for $\kappa$ means ranged from 2 to 15. To ensure that both $P_{mem}$ and $\kappa$ were in the correct parameter range, subject parameters for $P_{mem}$ were drawn from a normal distribution on the logit scale with a standard deviation of $\sigma=0.3$: $logit\left( P_{mem} \right)\sim N\left( logit\left( \mu\right),0.3 \right)$, and for $\kappa$ were drawn from a normal distribution on the log scale with a standard deviation of $\sigma=0.3$: $log\left( \kappa\right)\sim N\left( log\left( \mu\right),0.2 \right)$^^[[1]](#footnote-1)^^and then back-transformed to their native scale for generating the data for each subject.

The script for the parameter recovery study as well as all results reported here is also available via the GitHub repository containing all code examples: <https://github.com/GidonFrischkorn/Tutorial-MixtureModel-VWM>

**Analysis of parameter recovery**

As dependent variables of the parameter recovery, we calculated recovery of the hyper parameters (i.e. means for $P_{mem}$ & $\kappa$ in each simulated sample) via the correlation and the normalized root mean square error (RMSE) between the data generating hyper parameters and the estimated means form the ML and BMM approach on their native scale ($P_{mem}=\left[ 0,1 \right]$, $\kappa=\left[ 0,\infty\right]$). Additionally, we calculated recovery of subject level parameters via the correlation and RMSE between the data generating subject parameters and the estimated parameters from the ML and BMM approach for each simulation condition both for each repetition and aggregated across repetitions. The recovery of hyper parameters provides information how well suited the different approaches are to capture mean differences between samples or experimental condition, whereas the recovery of subject parameters gives a general assessment of parameter recovery of subject level parameters.

**Recovery of Hyper Parameters**

In Figure A1 you see the recovery of sample means over the 200 repetitions of the simulation for the probability of recalling items from memory ($P_{mem}$). Both the ML and BMM implementation recover the sample means quite well, although BMM seems to be slightly more accurate especially when $P_{mem}$ is smaller.

This is even more evident when considering the bias in the estimation of sample means as a function of the generating sample mean as shown in Figure A2. Here we can clearly see that estimating $P_{mem}$ with the ML implementation leads to overestimation of sample means if the true sample mean of $P_{mem}$ is small, whereas the Bayesian hierarchical estimation shows negligible bias over the whole range of simulated $P_{mem}$ sample means.

**Figure A1.** Recovery of sample means of $P_{mem}$ over the 200 repetitions run in each of the 12 simulation conditions.


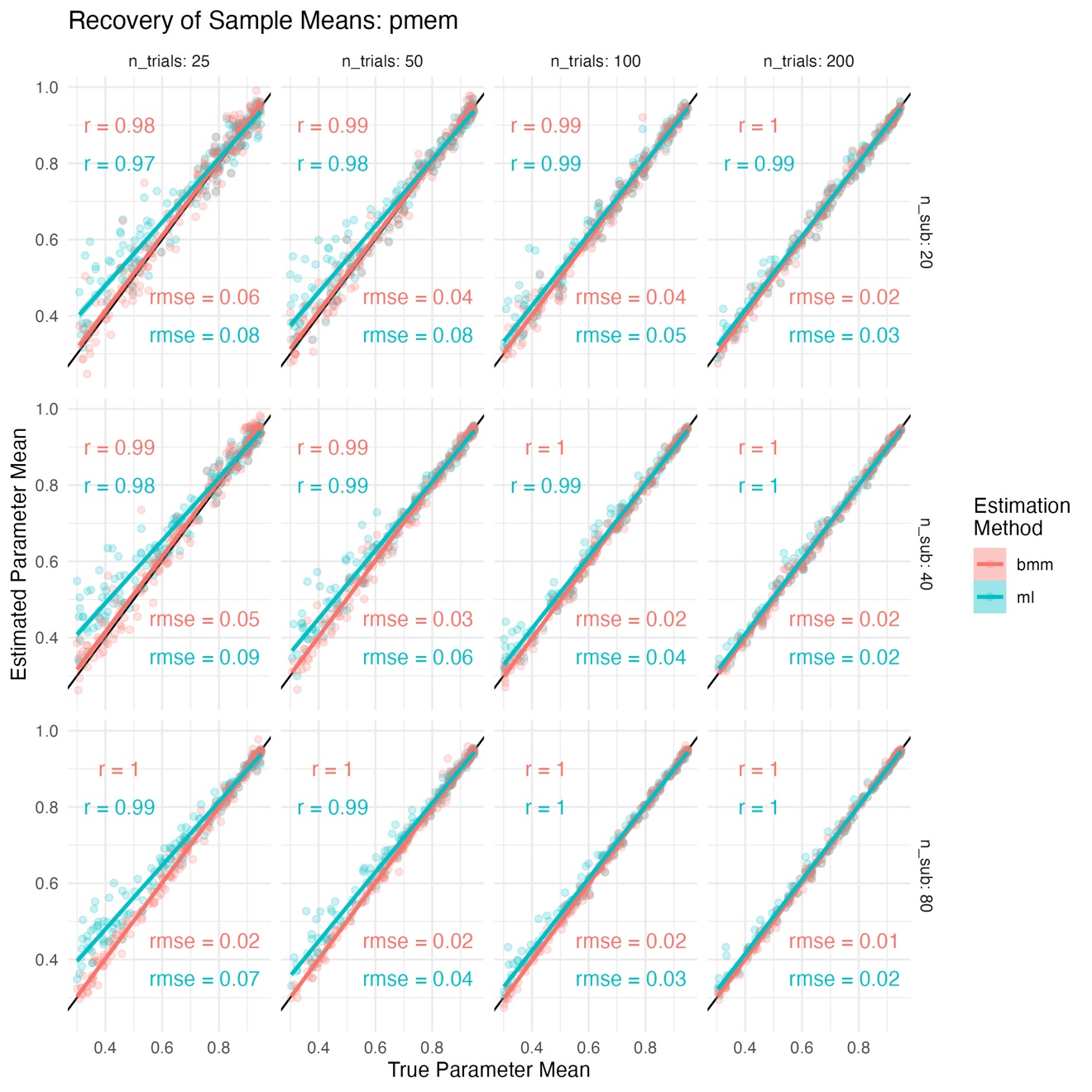


*Note.* Each point indicates one of the 200 repetitions with its unique true sample and estimated mean. The regression line illustrates a linear regression fit to the recovery data, and the black line provides a reference for a perfect and unbiased recovery of the true sample mean.

**Figure A2.** Bias in the estimation of sample means of $P_{mem}$ dependent on the sample mean generating the data.


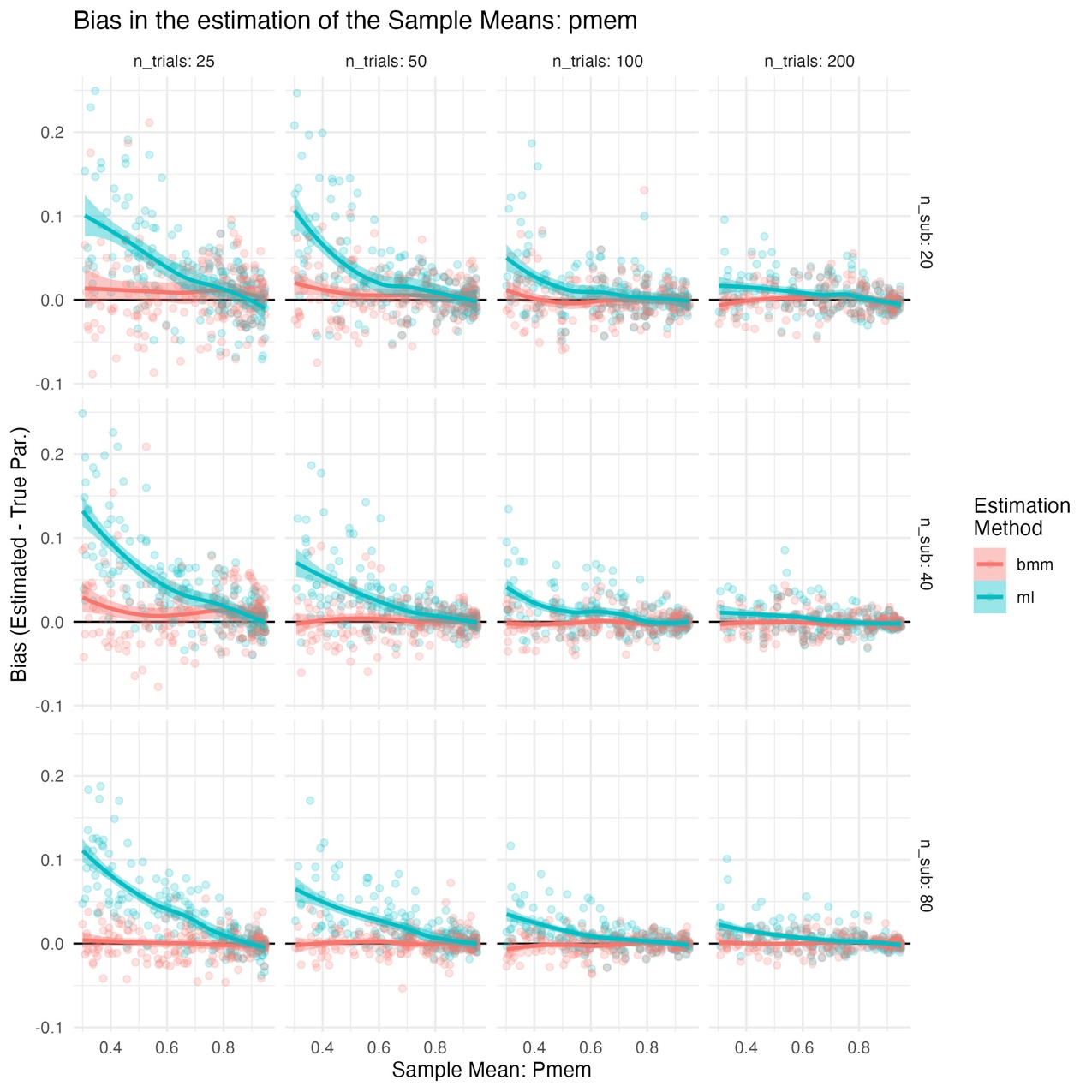


*Note.* Each point indicates the bias of one of the 200 repetitions. The regression line illustrates a local polynomial regression fit to the bias data with the black line as a reference for unbiased estimation.

For sample means of $\kappa$, the differences between estimation with subject-wise ML compared to hierarchical Bayesian estimation are much stronger than for $P_{mem}$. Figure A3 shows that the hierarchical Bayesian estimation implemented in bmm outperforms subject-wise ML, especially with less than 100 observations per subject. Although the rank ordering of the $\kappa$ sample means is still acceptable for more than 100 observations per subject for the ML approach, there are severe biases towards overestimating $\kappa$ with lower numbers of observations per participant that also compromise the recovery of rank order in the sample means. Although the Bayesian hierarchical approach is also slightly affected by the total amount of data used to estimate the sample mean of $\kappa$ it shows remarkably good recovery of sample means even with as little as 25 observations per subjects.

**Figure A3.** Recovery of sample means of $\kappa$ over the 200 repetitions run in each of the 12 simulation conditions.


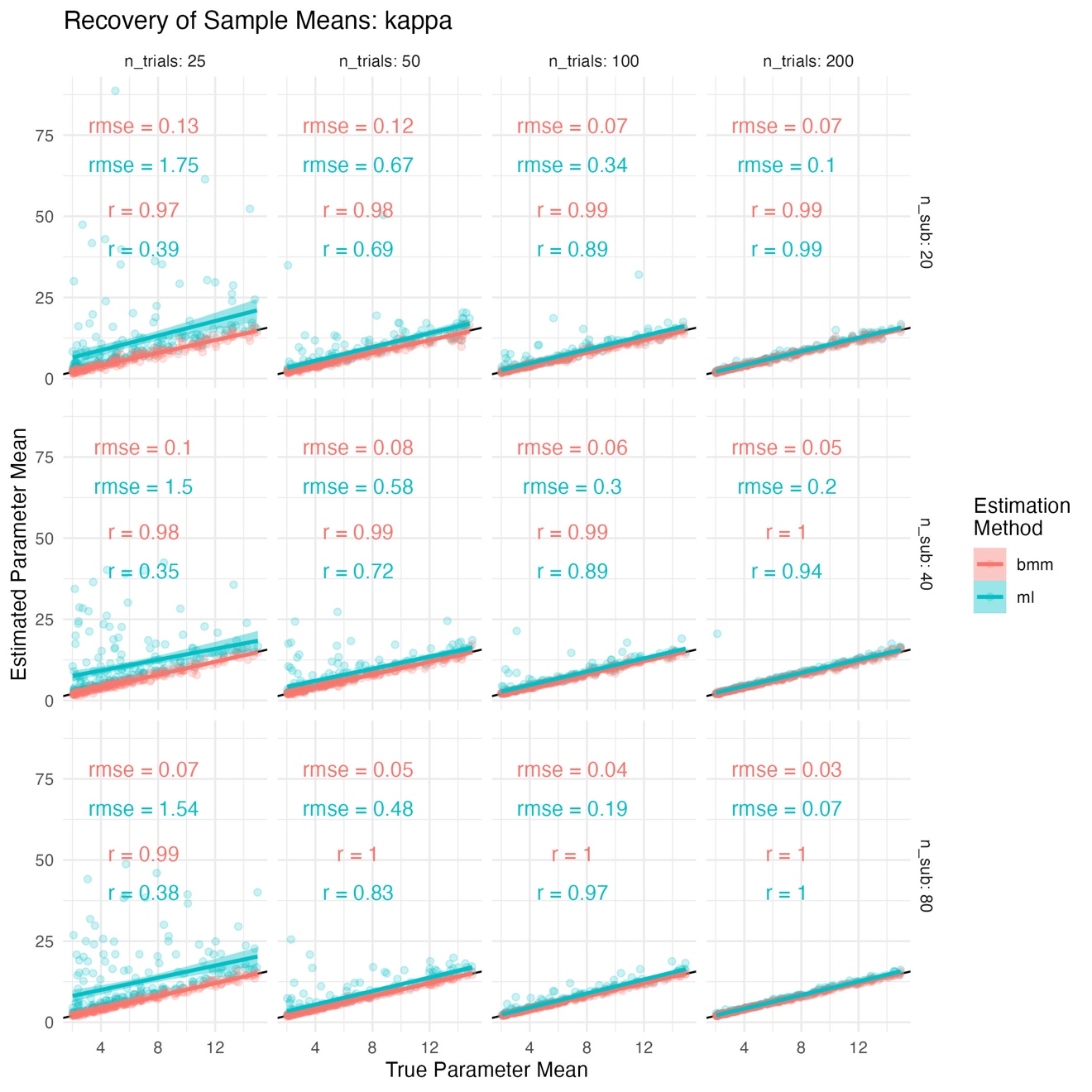


*Note.* Each point indicates one of the 200 repetitions with its unique true sample and estimated mean. The regression line illustrates a linear regression fit to the recovery data, and the black line provides a reference for a perfect and unbiased recovery of the true sample mean.

Previous recovery simulations have indicated that the recovery of $\kappa$ gets noisier as the proportion of recalling items from memory reduces and less data provides information on the precision of memory responses. Figure A4 illustrates that the subject-wise ML implementation indeed shows exactly this pattern when considering the estimation bias, whereas the hierarchical Bayesian implementations does not show this trend.

**Figure A4.** Bias in the estimation of sample means of $\kappa$ dependent on the sample mean generating the data.


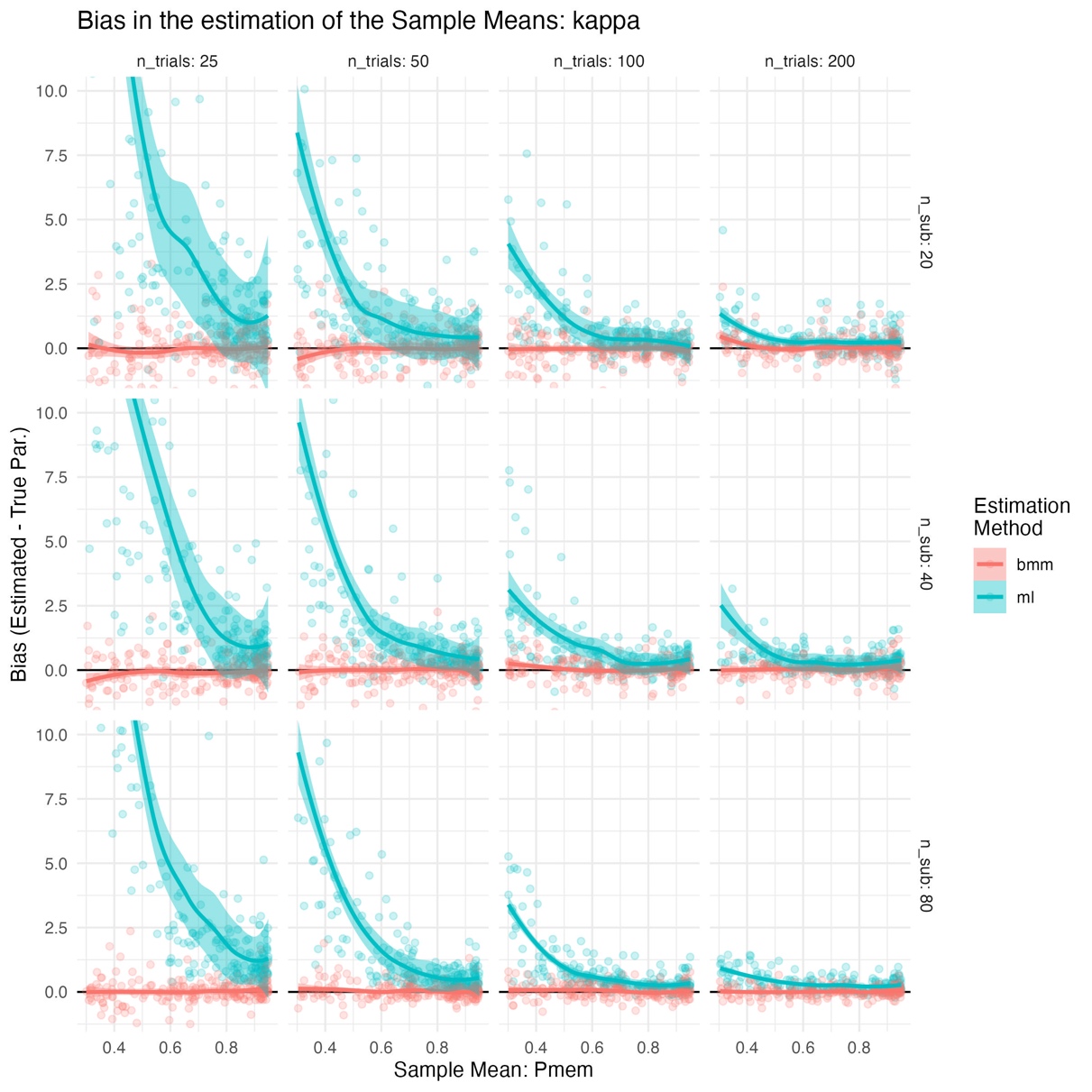


*Note.* Each point indicates the bias of one of the 200 repetitions. The regression line illustrates a local polynomial regression fit to the bias data with the black line as a reference for unbiased estimation.

**Recovery of Subject Parameters**

All in all, the recovery of subject parameters provides the same picture as the recovery of sample means. As would be expected, recovery was generally worse compared to the recovery of sample means, but still the hierarchical Bayesian implementation consistently outperformed the subject-wise ML estimation. Figure A5 illustrates the distribution of correlations of true with recovered subject parameters for both $P_{mem}$ and $\kappa$ for each repetition in the 12 conditions. It is evident that there is a lot of variability in the recovery, that is likely in part due to the smaller sample sizes and less variability between subjects compared to other simulations (e.g. Grange & Moore, 2022) and thus variability in the range of parameters (despite a constant standard deviation for generating subject parameters). However, to adequately gauge the estimation of sample means in experimental settings we consciously choose to simulate smaller samples.

The correlation estimates shown in Figure A5 obviously depend on the variability of parameters in the simulated sample. In our simulations this variability was considerably less than in other simulations (e.g. Grange & Moore, 2022) that covered the full range of reasonable parameter values. Instead, we choose to simulate subject parameters with smaller standard deviations that are likely to find in common experiments and samples (see for example: Souza et al., 2024).

**Figure A5.** Distribution of subject parameter recovery indicated by the correlation between true & estimated subject parameters in the 200 simulated samples in each of the 12 conditions.


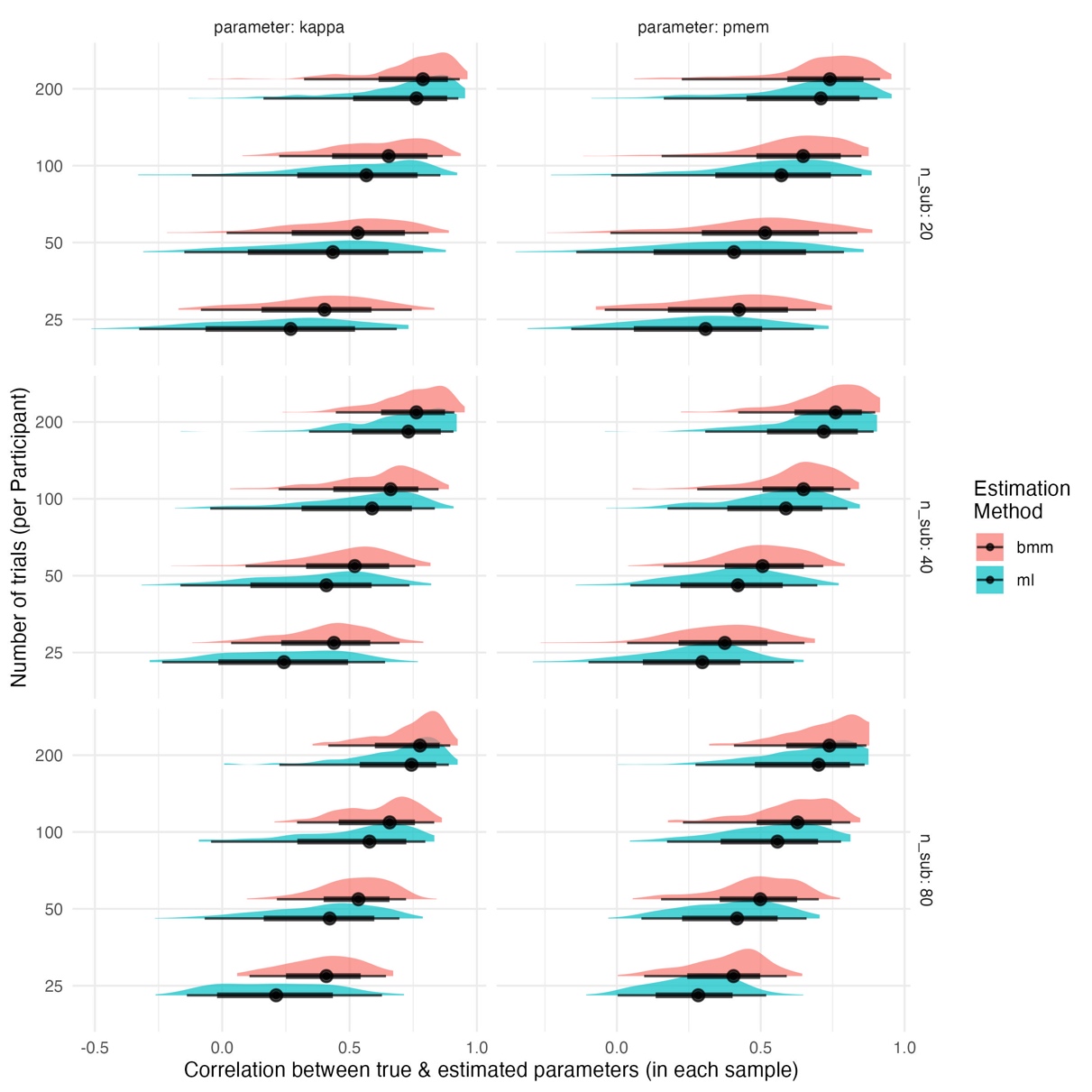


*Note.* The point at the bottom of the distribution indicates the median of the distribution, the thick line the middle 66% and the thin line the middle 95% of the distribution

A more independent indicator of recovery that does not depend on the standard deviation of the simulated sample is the RMSE. Figure A6 shows the RMSE of recovery of subject parameters. Here we can see that the hierarchical Bayesian estimation has consistently lower RMSE values even for very low number of observations per participant. In fact, subject-wise ML estimation occasionally has very large RMSE values especially for kappa when there is little data.

**Figure A6.** Distribution of subject parameter recovery indicated by the RMSE of estimated subject parameters in the 200 simulated samples in each of the 12 conditions.


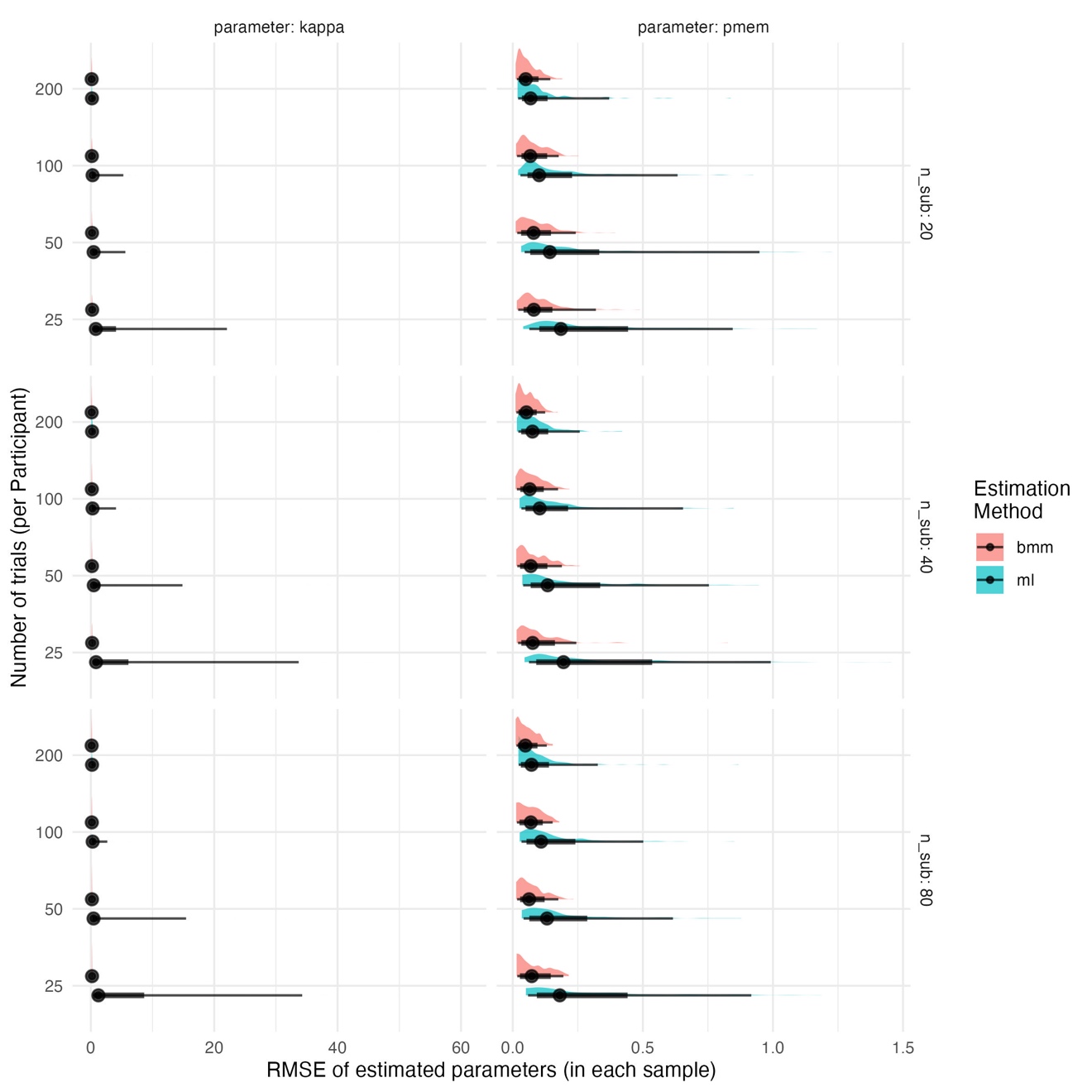


*Note.* The point at the bottom of the distribution indicates the median of the distribution, the thick line the middle 66% and the thin line the middle 95% of the distribution

As for the sample means, we also explored how recovery of subject parameter changes as the range of parameters differs. Figure A7 shows how the RMSE changes as a function of the sample mean of $P_{mem}$. The results indicate that the RMSE is smaller the larger the sample mean of $P_{mem}$, this effect is however stronger for the recovery with subject wise ML than for Bayesian hierarchical estimation.

**Figure A7.** RMSE of estimated $P_{mem}$ subject parameters dependent on the sample mean of $P_{mem}$.


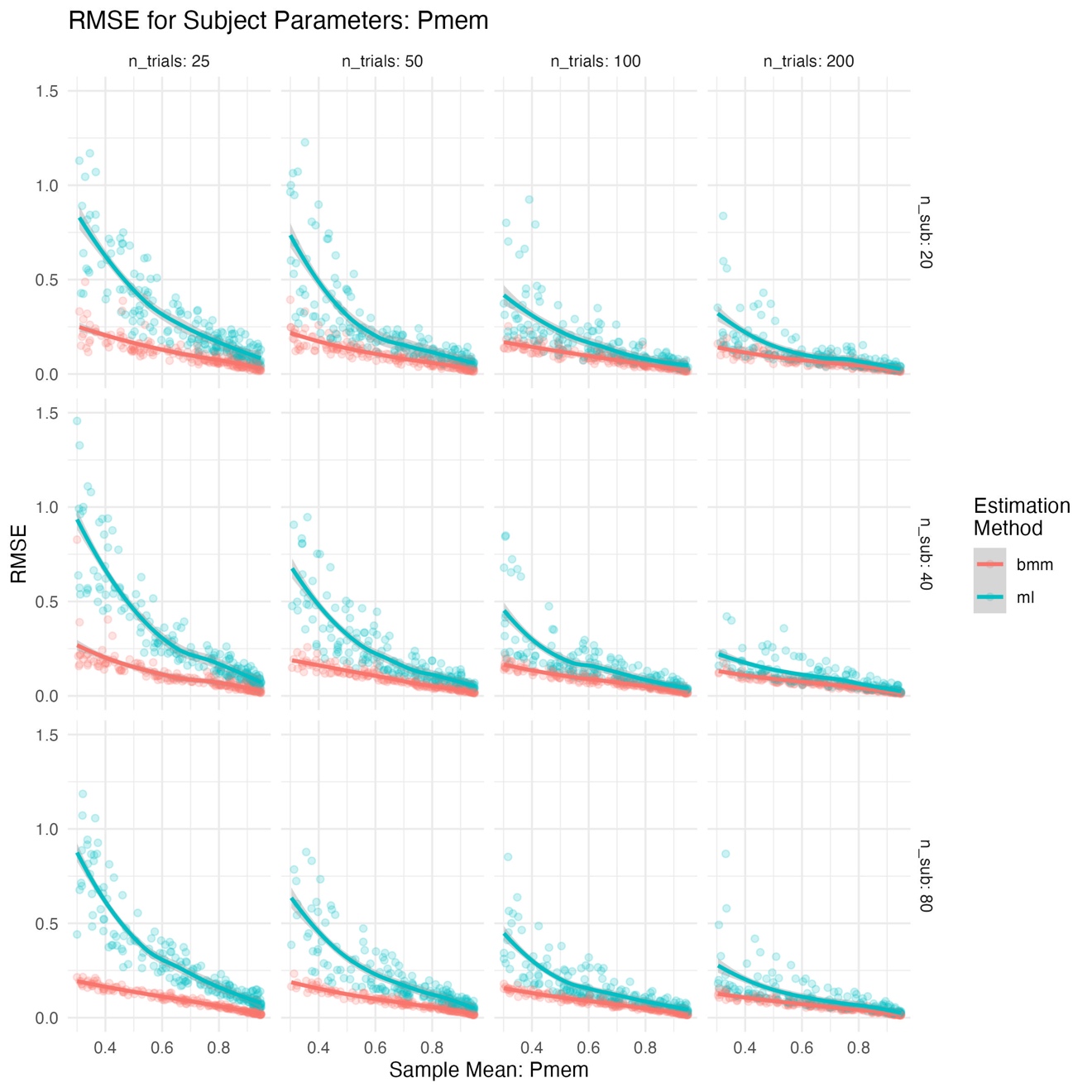


*Note.* Each point indicates the RMSE of subject parameter recovery in one of the 200 repetitions. The regression line illustrates a local polynomial regression fit to the bias data with the black line as a reference for unbiased estimation.

We find a similar pattern for the subject recovery of $\kappa$ as a function of the sample mean of $P_{mem}$, shown in Figure A8. For $\kappa$, however, the dependency of recovery on the sample mean of $P_{mem}$ is much stronger for subject-wise ML estimation, whereas the hierarchical Bayesian estimation shows almost no dependency of subject recovery of $\kappa$ by the sample mean of $P_{mem}$.

**Figure A8.** RMSE of estimated $\kappa$ subject parameters dependent on the sample mean of $P_{mem}$.


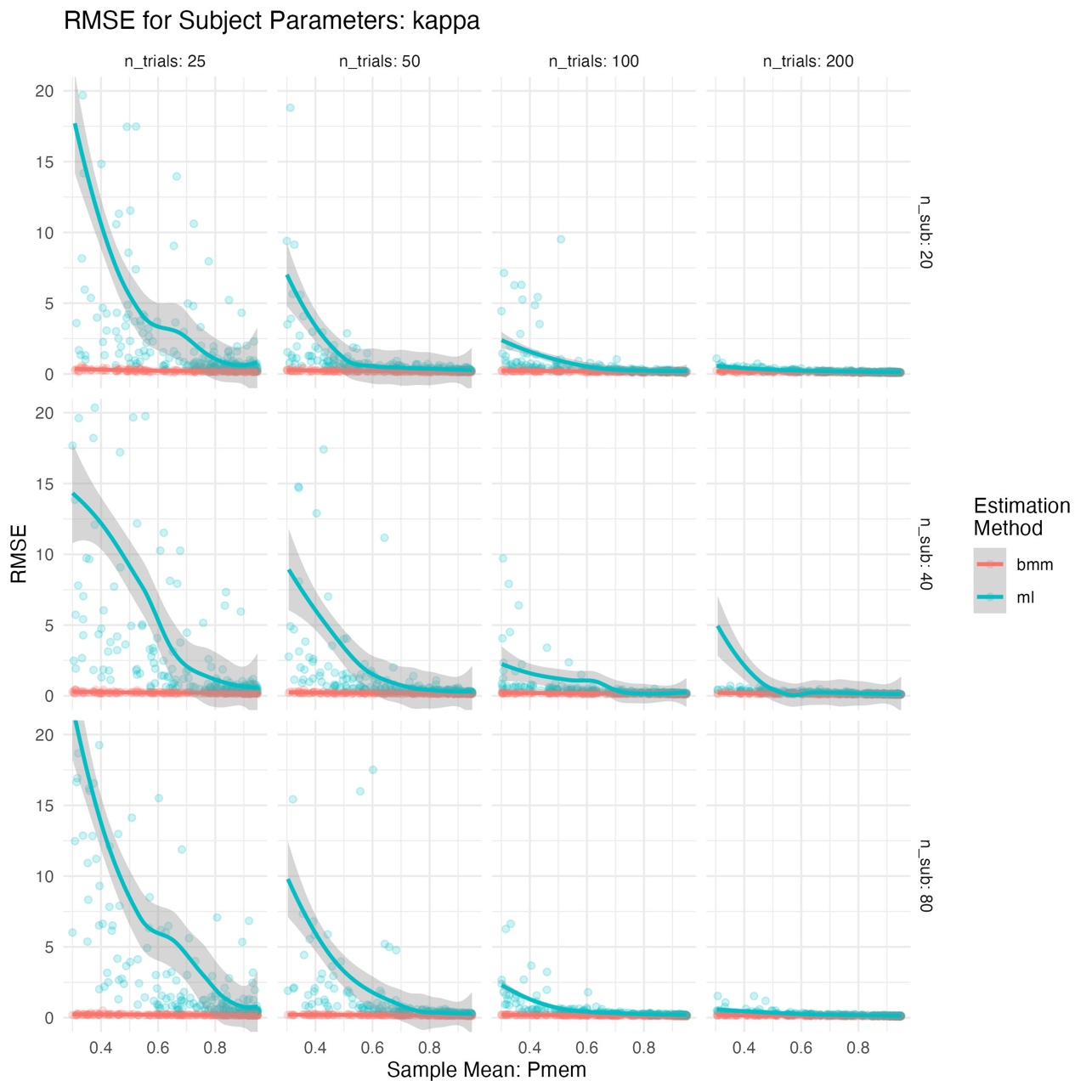


*Note.* Each point indicates the RMSE of subject parameter recovery in one of the 200 repetitions. The regression line illustrates a local polynomial regression fit to the bias data with the black line as a reference for unbiased estimation.

**Appendix B: Model comparisons for Example 5**

In this appendix, we present results of model comparisons for the different versions of the interference measurement model (IMM) and the three-parameter mixture model introduced in Example 5. We fitted all three versions of the IMM and the three-parameter mixture model to the data of Example 5 and ran 20 repetitions of a model comparison via Bayes Factors with these fitted models. The code for fitting all models and the Bayes Factor estimation is available in the Online Supplement on GitHub.

The results from these model comparisons (see Table B1) indicated evidence favoring the full IMM over all other models. However, the range of Bayes Factors also covers ambiguous evidence neither favoring the full IMM over the IMMabc or the three-parameter mixture model. This is likely due to the low number of posterior samples (8000) that these model comparisons are based on. The only conclusive comparison is that assuming that swaps occur solely due to the generalization of the context dimension as assumed by the IMMbsc are insufficient to explain the data, as this model is clearly not supported compared to any other model. Likewise, there is weak evidence favoring the three-parameter mixture model over the IMMabc, given that these models are mathematically equivalent this is surprising. However, these differences can arise due to different parametrizations, as has already been noted by Oberauer et al. (2017).

*Table B1*

Bayes Factors comparing the models in the top row against the respective model of each row. The top number in each cell indicates the median Bayes Factor over the 20 repetitions. The numbers in brackets below refer to the minimal and maximal Bayes Factor over all repetitions.

|  | IMM_full_ | IMM_abc_ | IMM_bsc_ |
| --- | --- | --- | --- |
| IMM_abc_ | 597.64  [1.45; 23.1*10^3^] |  |  |
| IMM_bsc_ | 4.7*10^39^  [8.48*10^35^; 1.77*10^42^] | 7.92*10^36^  [3.80*10^34^; 2.05*10^38^] |  |
| Mixture3p | 133.05  [0.41; 12.9*10^3^] | 0.22  [1.65 * 10^-3^; 31.42] | 2.81 * 10^-38^  [3.09*10^-40^; 1.47*10^-34^] |

1. The generation of subject parameters on the logit scale for $P_{mem}$ and on the log scale for $\kappa$ slighty favors the bmm implementation for parameter estimation in the hierarchical model, as these distributions are what is assumed as random effects on the parmaeter scale. However, we think these distributions over subject represent an adequate reflection of variation over subjects. [↑](#footnote-ref-1)
